# Gene expression identifies metabolic and functional differences between intramuscular and subcutaneous adipocytes in cattle

**DOI:** 10.1186/s12864-020-6505-4

**Published:** 2020-01-28

**Authors:** Nicholas J. Hudson, Antonio Reverter, William J. Griffiths, Eylan Yutuc, Yuqin Wang, Angela Jeanes, Sean McWilliam, David W. Pethick, Paul L. Greenwood

**Affiliations:** 1 School of Agriculture and Food Sciences, University of Queensland, Gatton, QLD Australia; 2 Agriculture, Commonwealth Science and Industrial Research Organisation, 306 Carmody Road, Brisbane, QLD Australia; 3 Swansea University Medical School, Swansea University, Singleton Park, Swansea, SA2 8PP Wales, UK; 4 Institute for Molecular Biosciences, University of Queensland, St. Lucia, Brisbane, QLD Australia; 5 School of Veterinary and Life Sciences, Murdoch University, Murdoch, WA 6150 Australia; 6 New South Wales Department of Primary Industries, Armidale Livestock Industries Centre, University of New England, Armidale, NSW 2351 Australia

**Keywords:** Bovine fat depots, Kidney, Omental, Intermuscular

## Abstract

**Background:**

This study used a genome-wide screen of gene expression to better understand the metabolic and functional differences between commercially valuable intramuscular fat (IMF) and commercially wasteful subcutaneous (SC) fat depots in *Bos taurus* beef cattle.

**Results:**

We confirmed many findings previously made at the biochemical level and made new discoveries. The fundamental lipogenic machinery, such as *ACACA* and *FASN* encoding the rate limiting Acetyl CoA carboxylase and Fatty Acid synthase were expressed at 1.6–1.8 fold lower levels in IMF, consistent with previous findings. The FA elongation pathway including the rate limiting *ELOVL6* was also coordinately downregulated in IMF compared to SC as expected. A 2-fold lower expression in IMF of *ACSS2* encoding Acetyl Coenzyme A synthetase is consistent with utilisation of less acetate for lipogenesis in IMF compared to SC as previously determined using radioisotope incorporation. Reduced saturation of fat in the SC depot is reflected by 2.4 fold higher expression of the *SCD* gene encoding the Δ9 desaturase enzyme. Surprisingly, *CH25H* encoding the cholesterol 25 hydroxylase enzyme was ~ 36 fold upregulated in IMF compared to SC. Moreover, its expression in whole muscle tissue appears representative of the proportional representation of bovine marbling adipocytes. This suite of observations prompted quantification of a set of oxysterols (oxidised forms of cholesterol) in the plasma of 8 cattle exhibiting varying IMF. Using Liquid Chromatography-Mass Spectrometry (LC-MS) we found the levels of several oxysterols were significantly associated with multiple marbling measurements across the musculature, but (with just one exception) no other carcass phenotypes.

**Conclusions:**

These data build on our molecular understanding of ruminant fat depot biology and suggest oxysterols represent a promising circulating biomarker for cattle marbling.

## Background

Deposition of marbling (IMF) fat in cattle is commercially valuable. It has a positive impact on organoleptic properties of meat such as flavour, juiciness and tenderness [1]. On the other hand, the other fat depots including subcutaneous and organ fats do not add value to meat cuts. Excessive amounts of these undesirable depots are often associated with carcasses expressing high levels of IMF. Therefore, there is a continued interest in developing our understanding of the metabolic and functional differences between the various fat depots with a view to better uncouple IMF and SC deposition.

Marbling has been considered a late maturing trait only becoming visible after the other depots, this despite relative rates of increase in IMF being similar to other fat depots [2–4]. Furthermore, while breeds of cattle like Wagyu and Hanwoo are pre-disposed to precociously and selectively develop IMF, the underlying genetic, cellular, biochemical and physiological mechanisms have not been well established [5]. We know from previous work that marbling adipocytes tend to be relatively small [6] and comprising more saturated fatty acids [7] compared to those in the SC depot. Developmentally, marbling adipocytes are thought to arise from differentiation and lipid filling of fibroblasts within perimysial connective tissue [8].

In terms of differential metabolism between depots, previous biochemical evidence points to IMF having relatively slow rates of lipogenesis in both cattle [9] and pigs [10] and under certain nutritional circumstances a substrate preference for glucose carbon over acetate when compared to SC [6, 11]. Post-weaning diets tailored to these specific metabolic properties of IMF, such as strategic feeding with high energy concentrate, have had mixed success [12] for reasons not certain but which probably include net energy available for tissue deposition. A recent review emphasises castration, digestion and absorption of feed, glucose availability and vitamin A, D and C levels as important factors in marbling development [13]. However, overall it is clear that there is scope for a deeper understanding of ruminant fat depot metabolism and biology that may inform new animal management strategies.

The emergence of genome-wide transcriptome screening technologies provides an opportunity to assess entire biochemical pathways in quantitative detail not yet possible at other levels of biological organisation. Here, we analyse data from 5 bovine fat depots (IMF, SC, intermuscular, kidney and omental), with a particular focus on the IMF versus SC depot comparison. These functional genomic data are one component of a much larger animal experiment exploring cattle genotype by nutritional effects on fat depot biology [12]. Tissue samples for the present study were taken from 26 month-old steers of 3 genotypes, Angus, Hereford and Wagyu x Angus following high energy nutrition in a feedlot for 259 days. The Herefords had relatively low IMF and high SC whereas the Angus and Wagyu x Angus were higher IMF and lower SC.

We explored two analytical approaches both focussing on differential fat depot biology, but with one hypothesis-driven and one hypothesis-free. The former tested the mRNA expression of canonical ruminant fatty acid synthesis and degradation pathways and compared the output against prior biochemical expectation. The latter explored genome-wide patterns of gene expression with an aim of making new metabolic and functional discoveries in an unbiased manner. The across depot genome-wide transcriptome data submitted with this research article represents a uniquely powerful data resource within the field of ruminant fat biology.

## Results

### Hypothesis-free screen

#### Data driven hierarchical clustering

**Fig. 1:**
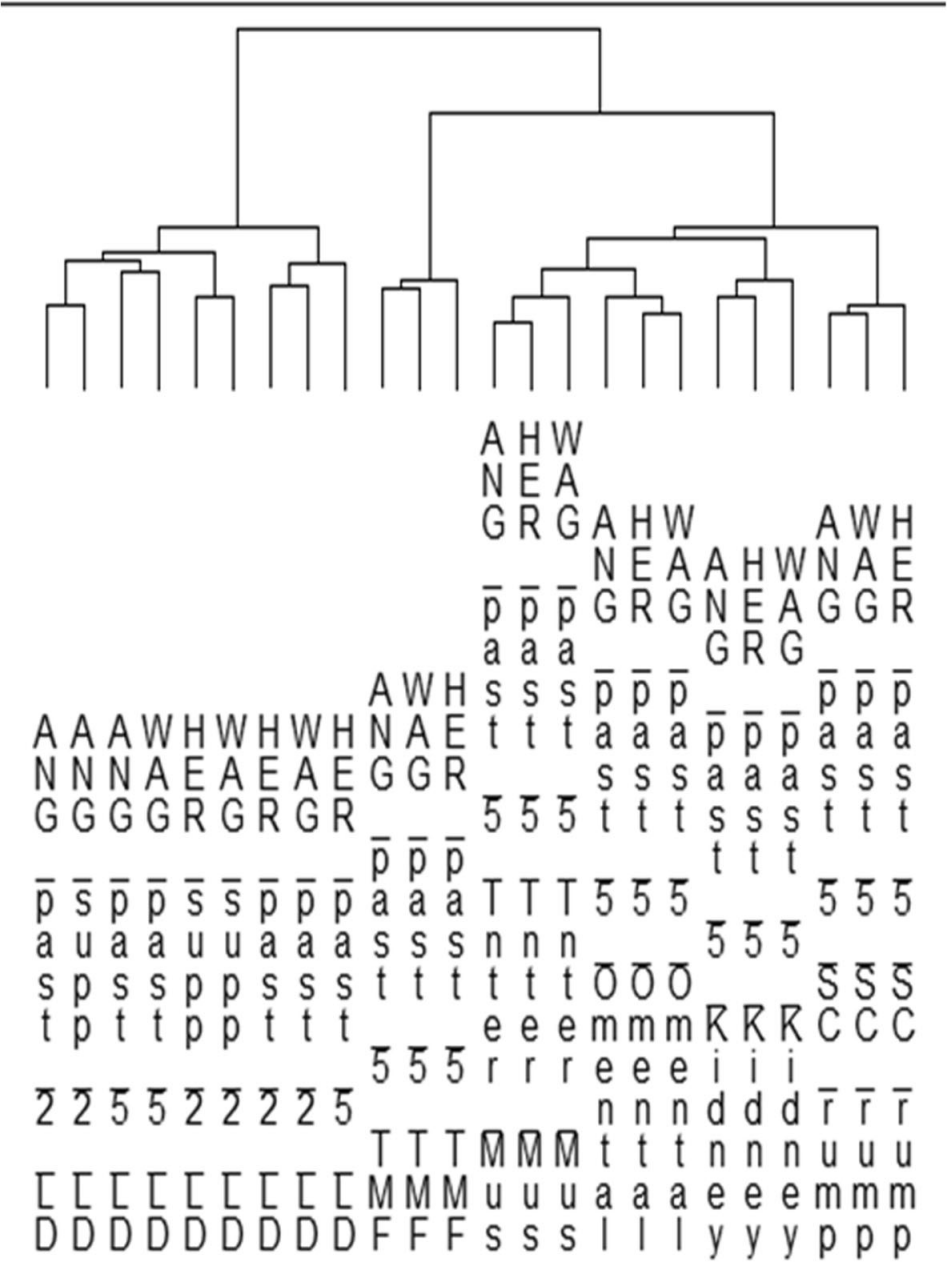
A dendrogram of relationships between the various fat depots based on the expression profiles of 10,000 genes selected at random. The treatment labels are breed (Ang = Angus, Her = Hereford, Wag = Wagyu x Angus), diet (past = pasture, supp = supplement), Kill number (2 or 5) and finally tissue (LD = longissimus dorsi muscle, IMF = intramuscular fat, Inter Mus – intermuscular fat, Omental = omental fat, Kidney = kidney fat, SC rump = subcutaneous rump fat). The first major split shows that the LD muscle is discriminated from all fat depots, reflecting muscle-specific patterns of gene expression. All fat depots are clearly resolved i.e. the three breeds form fat depot specific clusters in all cases which shows the various depots all possess diagnostic genome-wide expression signatures. IMF was awarded a unique branch within the fat tree, but this is presumably influenced by muscle derived gene expression arising from small amounts of LD muscle contamination

Each fat depot could be clearly discriminated by gene expression as the data from each breed clustered at the depot level (Fig. 1). Put another way, the gene expression differences between fat depots clearly overwhelm any breed differences within a depot. Moreover, IMF was separated from the other 4 fat depots. Of the remaining 4 depots, Inter and Omen were most closely related, followed by Kid then SC. Given SC appears the most functionally divergent of the ‘pure’ (i.e. we can make a confident assertion of no muscle contamination) fat depot samples, we elected to compare all depots to SC in turn.

#### Differential expression (DE) analysis

##### SC versus all other fat depots

**Fig. 2:**
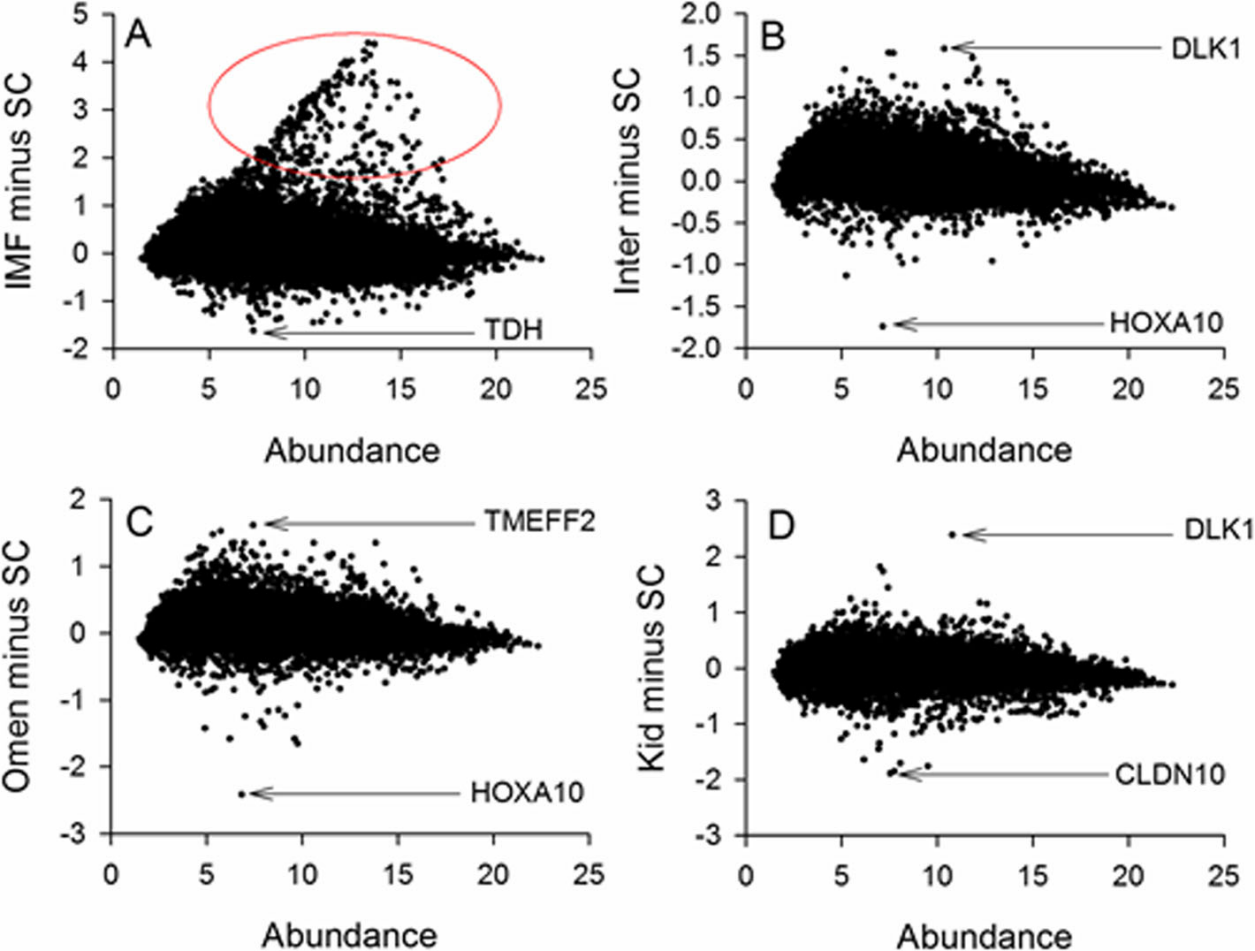
The Minus Average (MA) plots of SC versus all other depots (**a**) IMF (**b**) Inter (**c**) Omen and (**d**) Kid. It is clear that SC tends to have relatively high expression of *HOXA10* and relatively low expression of *DLK1*. The red circle in (**a**) denotes an unusual triangular shaped protuberance atypical of MA plots and whose functional characteristics were subsequently explored

We plotted all fat depots minus SC and annotated the extreme differentially expressed (DE) genes (Fig. 2). Two consistent outlier genes by expression profile in SC are *HOXA10* and *DLK1* (*P* < 0.01). The log2 normalised mean expression of these two genes in each fat depot is compared in Table 1. Other genes of interest for their extreme expression in at least one depot are *TDH*, *TMEFF2* and *CLDN10,* also tabulated in Table 1.

**Table 1:** Genes highly divergently expressed in SC versus all depots (log2 normalised mean expression). *P* values are reported for both DE and PIF (based on the SC versus IMF comparison)

Gene	Probe	LD	IMF	SC	Inter	Kid	Omen	Function	*P* value (DE / PIF)
*HOXA10*	A_73_104882	6.99	6.94	8.03	6.29	7.34	5.61	Differs among undifferentiated pre-adipocytes between depots	0.000265 / 0.013
*DLK1*	A_73_P069591	11.84	11.27	9.58	11.16	11.97	10.54	Pre-adipocyte factor	0.00000185 / 0.0000225
*TDH*	A_73_P042901	5.99	6.49	8.11	7.61	7.29	7.53	Catalyses the conversion of threonine to pyruvate	0.000000276 / 0.000919
*TMEFF2*	A_73_109150	7.43	7.48	6.61	7.58	6.96	8.22	Membrane protein associated with neurons	0.0125 / 0.101
*CLDN10*	A_73_106401	6.23	7.42	8.68	7.69	6.82	7.28	Claudin membrane protein associated with adhesion and ion transport	0.0000376 / 0.00343

We next focussed on the particular IMF versus SC comparison in more detail.

##### IMF versus SC

In the IMF versus SC comparison the most extreme 1% (145 out of 14, 476) downregulated genes in IMF enriched for ‘humoral immune response’ (Hypergeometric statistic, FDR q-value = 0.00017) based on the presence of genes including *CFB*, *CXCL3, CD163, CD36* and *CD163L1*). To generate this ranked list we used a modified DE metric called Phenotypic Impact Factor (PIF), which is a product of DE and average abundance across the treatments of interest. The extreme 20 most downregulated genes are shown in Table 2.

**Table 2:** The 20 most downregulated genes in IMF versus SC (log2 normalised mean expression). *P* values are reported for both DE and PIF (based on the SC versus IMF comparison)

Gene	Probe	LD	IMF	SC	Function	*P* value (DE / PIF)
*SCD*	A_73_P252739	14.46	15.33	16.47	Desaturation, fatty acid synthesis (oleic acid)	0.000153 / 0.00000163
*TF*	A_73_109609	9.57	11.04	12.47	Iron transport	0.00000421 / 0.00000731
*CXCL3*	A_73_108688	11.34	12.09	13.36	Secreted growth factor, inflammation	0.0000332 / 0.0000151
*DGAT2*	A_73_118582	14.51	15.86	16.72	Final reaction in TAG synthesis	0.00259 / 0.000132
*PCK2*	A_73_P102501	12.44	13.49	14.44	Adipogenesis, mitochondrial	0.00112 / 0.0003
*RAB9B*	A_73_104413	12.83	14.19	15.09	Endosome to golgi transport, membrane trafficking	0.00180 / 0.000326
*CFB*	A_73_118840	10.91	11.98	12.98	Complement factor B	0.000681 / 0.000528
*FASN*	A_73_P174332	16.08	17.23	17.94	Synthesis of long chain saturated fatty acids	0.00918 / 0.000528
*ACSS2*	A_73_P037091	12.58	13.11	14.01	Activation of acetate for lipid synthesis	0.00180 / 0.000670
*TDH*	A_73_P042901	5.99	6.49	8.11	Catalyses l-threonine degradation	0.00117 / 0.000919
*CD163*	A_73_P091466	7.18	7.81	9.20	Inflammation, strongly expressed by macrophages	0.00000719 / 0.000928
*G0S2*	A_73_100624	13.72	15.16	15.91	FA, TAG and ketone metabolism	0.00666 / 0.00106
*TIMP4*	A_73_112376	14.43	14.97	15.73	Inhibitor of matrix metalloproteinases	0.00614 / 0.00105
*AGPAT2*	A_73_118412	17.80	18.26	18.89	De novo phospholipid synthesis, endoplasmic reticulum	0.0168 / 0.00102
*CCDC80*	A_73_P035251	15.24	16.08	16.79	Extracellular matrix	0.00918 / 0.00119
*QPRT*	A_73_P039661	11.69	12.80	13.62	NAD de novo biosynthesis	0.00370 / 0.00204
*SPARC*	A_73_P300606	17.10	17.85	18.43	Extracellular matrix organisation	0.0239 / 0.00258
*CIDEA*	A_73_100290	12.16	12.84	13.64	Regulation of lipolysis	0.00439 / 0.00245

From Table 2 it can be seen that various aspects of fat metabolism (*SCD, DGAT2, FASN, ACSS2, AGPAT2, CIDEA, G0S2*), extracellular matrix biology (*TIMP4, SPARC, CCDC80*) and some inflammation related genes (CXCL3, CD163) are prominently featured in those transcripts whose expression is lower in IMF than SC. The extreme 5% downregulated genes (PIF) in IMF enriched for ‘lipid metabolic process’ (FDR q*-*value = 1.04 e-16) and ‘defense response’ (FDR q*-*value 1.88 e-15) in line with those observations, but these functional enrichments were not as extreme as the top hit ‘regulated exocytosis’ (FDR q*-*value = 5.11 e-27).

On the other hand, the upregulated 1% in IMF enriched very significantly for ‘muscle system process’ (FDR q-value = 3.54 e-46) based on 44 genes generally regarded as either muscle-specific (e.g. myoglobin) or very highly expressed in muscle cells (such as numerous specialised myosin light and heavy chain isoforms as illustrated by *MYL2* and *MYH2*). A more lenient 5% extreme upregulated PIF (or 724 genes) marginally lessens the impact of the muscle specific detection (FDR q-value = 3.44 e-44).

It is clear from these functional enrichments that the dissected IMF must have contained a very small amount of longissimus dorsi (LD) muscle contamination not present in the other fat depots. Genes strongly expressed by the muscle cells in the IMF sample therefore appear to make up the majority of the atypical triangle shaped protuberance in the IMF versus SC MA plot (as highlighted by the red circle on Fig. 2a). We have elected not to tabulate the extreme upregulated genes in IMF versus SC, as this would simply return a list of genes dominated by mRNA encoding muscle structural and muscle metabolic proteins.

**Fig. 3:**
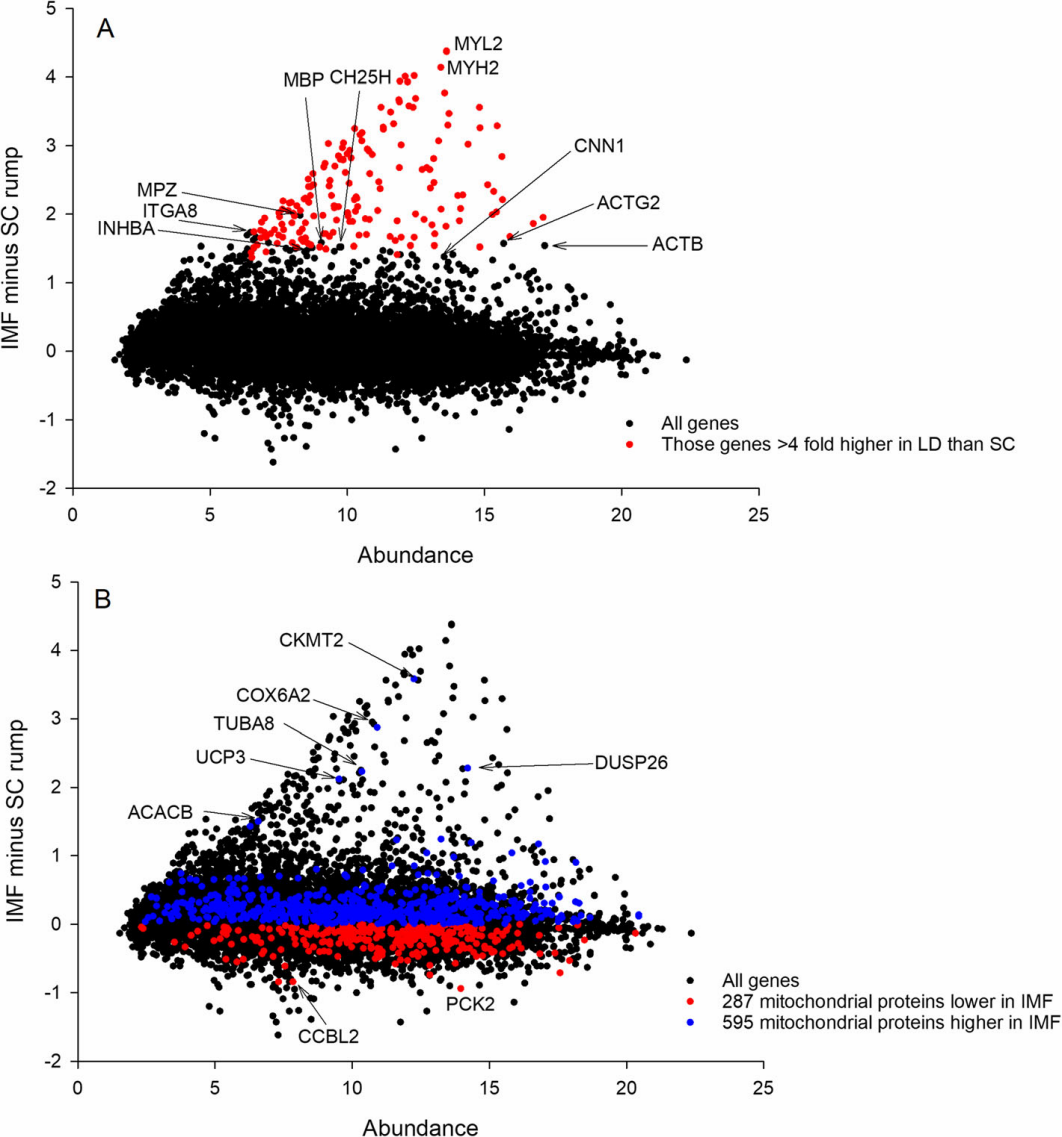
Minus Average (MA) plots of IMF minus SC. **a** Red dots are those 168 mRNA 4 fold higher in LD than SC indicating the atypical triangular protuberance comes from contaminating LD (**b**) the 886 mRNA encoding mitochondrial proteins for which we detected matches in our data, blue above 0, red below 0. There is a significant skew upwards (595 above the 0 line) from the null expectation of equilibrium (data centred on 0, with 443 falling on either side of the line)

To better determine the cellular origin (marbling adipocyte versus myocyte) of many of the upregulated genes in the dissected IMF we first computed Differential Expression (DE) between pure LD (nearly all muscle, small amount of IMF) and SC (all fat, no muscle contamination). We then identified genes which had nominally > 4 fold higher expression in LD than SC as likely being transcribed predominantly from muscle cells, not fat cells. This yielded a list of 171 genes (hypergeometric FDR q value of 2.04e-39 for “muscle system process”), which we have then highlighted on the IMF versus SC plot (Fig. 3 panel a**;** Additional file 1). The highlighted genes, which have been identified using a numerical strategy, all clearly fall in the triangular shaped protuberance distorting the overall IMF versus SC MA distribution, indicating that this atypical data distribution is indeed a consequence of muscle contamination.

This set of analyses reinforces the conclusion that prima facie those outlier genes in the IMF versus SC are almost certainly driven by the presence of some LD muscle in the ‘pure’ IMF sample and therefore need to be interpreted cautiously. The impact of the ‘contaminating’ muscle derived RNA on the expression of most of the remaining genes is harder to foresee, as there will be a continuum of shared expression between the myocytes and adipocytes, depending on the particular gene being investigated. In this submission, we have provided the normalised mean expression for LD in addition to all the fat depots for the 34,227 probes (Additional file 2) so the interested reader can assess the possible impact of contaminating LD on a case by case basis. This is also the reason why we have included the LD normalised mean expressions in each table for comparison. The outcome of a multiple criteria thresholding process is described below (*“Structural differences between marbling adipocytes and the other fat depot adipocytes”*). This multiple thresholding does allow us to define lists of genes whose expression likely arises from the IMF adipocytes themselves and therefore can be considered biologically informative of the IMF depot.

##### Mitoproteome

In an effort to explore the behaviour of the mitochondria in our understanding of the metabolism of IMF versus SC metabolism we quantitated the collective expression of those mRNA known to encode mitochondrial proteins. Using the downloaded mitochondrial protein database we matched 886 of these in our data, 595 of which were higher in IMF than SC, but only 287 of which were lower (Fig. 3 panel b). This is a significant deviation (*P* = 2.29 e-26) from the null expectation of equilibrium (i.e. symmetrically distributed around 0, with 443 above and 443 below) assessed by binomial distance. This upward skew is consistent with IMF having a higher mitochondrial content and / or mitochondrial activity than SC, but the presence of some high mitochondrial content LD muscle in the IMF depot is presumably influencing the result. Notable among the mitochondrial genes strongly downregulated in IMF compared to SC (despite the probable impact of the contaminating muscle) is *PCK2*.

##### Structural differences between marbling adipocytes and the other fat depot adipocytes

**Fig. 4:**
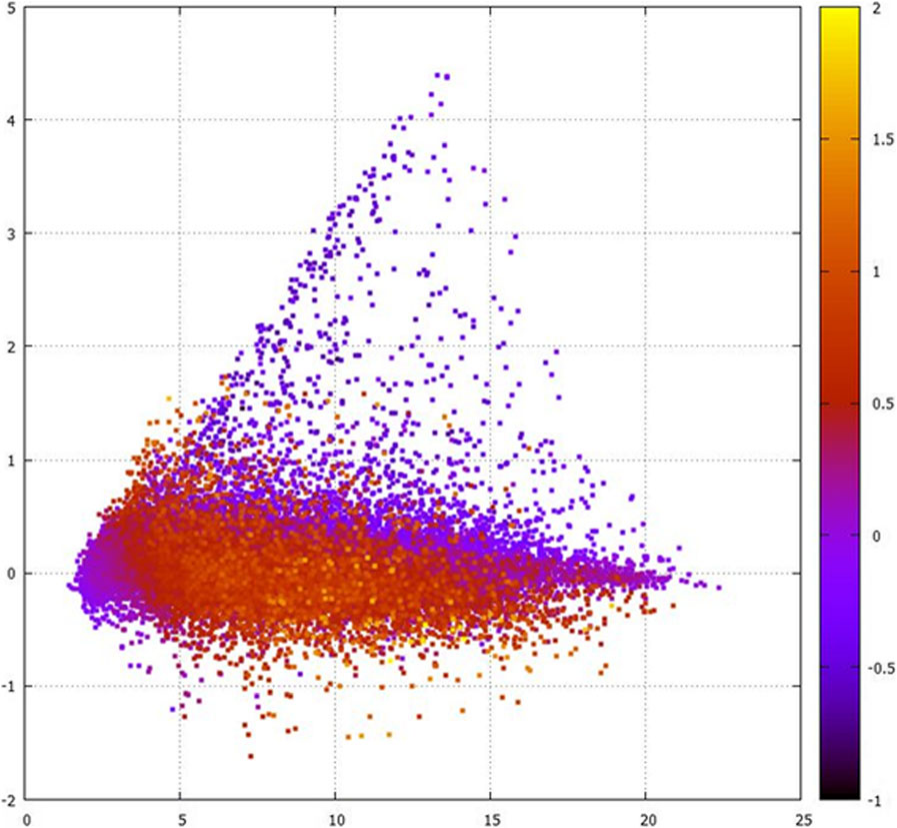
Modified Minus Average (MA) plot of IMF versus SC to allow the visualisation of the real IMF signal, independent of the LD contamination. Here, we have colour coded each mRNA using a formal numerical approach such that the expression of those highlighted in red and yellow tones (above 0) and purple (below 0) are most likely derived from the IMF adipocytes themselves and not from contaminating LD muscle. To achieve the colour coding we exploited the difference in expression detected between the ‘pure’ IMF and ‘pure’ LD muscle samples. For example, if an mRNA has higher expression in IMF than in LD then we conclude it likely derives from the marbling adipocytes. The mRNA encoding *CH25H* exemplifies this logic. In addition to being higher in IMF than SC, it is also higher in IMF than LD. In terms of exact position on the plot, the mRNA encoding *CH25H* has an A value of 9.77 and an M value of 1.52

To better account for and visualise the effect of the contaminating LD on the IMF gene expression we colour coded the Minus Average (MA) plot comparing IMF to SC based on a formal numerical analysis that accounts for the presence of contaminating LD in the IMF sample (Fig. 4). Colour coding each gene individually in this manner visually highlights those genes (such as *CH25H*) that are most likely higher in IMF than SC because of particularly high expression from the IMF adipocytes per se and not because of the contaminating LD muscle. Yellow / orange dots above 0 on the y axis are colour coded in this manner because they have a higher expression in IMF than LD which implies their observed higher expression in IMF than SC is a true feature of the marbling adipocytes. This logic also applies to the purple dots below 0 on the y axis.

To identify a short list of these genes whose high expression is confidently ascribed to the marbling adipocytes and not contaminating skeletal muscle we used a multiple criteria thresholding approach. To begin with, we asked the question, “which mRNA are more highly expressed in IMF than the average of all other fat depots by <1.32 fold (a difference of 0.4 on the log2 scale) and also much more highly expressed in IMF than LD (>2 fold).” This analysis returns a list of 49 genes whose expression is more confidently derived from marbling adipocytes (Additional file 1). There is no functional enrichment for ‘muscle systems process.’ Tabulating the top 20 of these ranked on IMF SC Phenotypic Impact Factor (PIF) yields the gene list in Table 3.

**Table 3:** Genes more highly expressed in IMF than all other fat depots by at least 1.32 fold whose expression appears driven by marbling adipocytes (IMF expression greater than 2 fold higher than LD). Normalised expression data expressed as log2 values. *P* values are reported for both DE and PIF (based on the SC versus IMF comparison)

Gene	Probe	LD	IMF	SC	Inter	Kid	Omen	Function	*P* value (DE / PIF)
*CNN1*	A_73_116127	12.93	14.22	12.84	13.20	12.98	12.88	Calponin 1, cytoskeleton	0.0000974 / 0.00000727
*ACTA2*	A_73_102355	15.75	16.79	15.69	15.96	15.88	15.78	Actin alpha 2, cytoskeleton	0.00180 / 0.0000175
*MYH11*	A_73_103577	13.12	14.33	13.03	13.31	13.16	13.06	Myosin heavy chain 11, cytoskeleton	0.000239 / 0.0000191
*ACTB*	A_73_P082186	10.63	12.12	10.64	11.09	10.85	10.68	Actin beta, cytoskeleton	0.0000295 / 0.000000000212
*CH25H*	A_73_113925	9.18	10.53	9.01	9.83	9.69	9.82	Cholesterol 25 hydroxylase, inflammation, lipid metabolism	0.0000179 / 0.000334
*ACTRT2*	A_73_107737	7.99	9.25	7.78	8.26	8.04	8.40	Actin related protein T2	0.0000334 / 0.00230
*INHBA*	A_73_108840	8.04	9.18	7.72	8.27	7.94	8.32	Inhibin A, Growth and Differentiation Factor, Hormone, binds ACVR2A	0.0000377 / 0.00283
*SORBS2*	A_73_P046131	10.85	12.12	11.06	11.27	11.15	11.27	Sorbin and SH3 domain containing. Cytoskeleton, lipid raft interaction	0.0026 / 0.00301
*NDRG4*	A_73_109411	9.76	10.80	9.63	9.92	9.91	9.82	Many developmental processes, ERK signalling, plasma membrane	0.000922 / 0.00352
*TNC*	A_73_108252	8.03	9.22	7.83	8.67	8.09	7.87	Tenascin, extracellular matrix	0.0000867 / 0.00379
*RAMP3*	A_73_P043356	6.56	7.92	6.34	7.03	6.69	6.81	Trans-membrane, transports calcitonin receptor-like protein	0.00000824 / 0.00577
*TNMD*	A_73_110381	6.16	7.19	5.51	6.45	5.34	5.33	Tenomodulin, genetic variants associated with type II diabetes	0.00000212 / 0.00829
*KRT18*	A_73_118812	5.99	7.39	5.77	6.04	5.83	6.68	Keratin 18, cytoskeleton	0.00000484 / 0.0087
*CTPS2*	A_73_113928	13.04	14.25	13.55	13.63	13.46	13.50	CTP synthase 2, rate limiting enzyme for CTP production from UTP.	0.0404 / 0.0157
*TAGLN*	A_73_P291026	9.36	10.43	9.51	9.90	9.67	9.72	Transgelin, actin cross-linking	0.0085 / 0.0186
*GJA5*	A_73_P048871	9.66	10.69	9.80	10.20	10.23	10.23	Connexin gene family, plasma membrane	0.0107 / 0.0147
*DKK3*	A_73_116904	10.33	11.58	10.77	11.13	11.12	10.96	Wnt signalling, many developmental processes	0.0194 / 0.0236
*MKX*	A_73_105199	5.25	6.34	4.97	5.75	4.96	5.11	Collagen biosynthesis	0.000109 / 0.0490
*MIR145*	A_73_105615	12.31	13.61	13.04	13.07	12.96	13.01	microRNA, little known	0.0857 / 0.0515
*COL28A1*	A_73_107519	4.90	6.28	4.93	5.42	5.49	5.05	Extracellular matrix	0.0137 / 0.0540

In this adapted list whose expression signals are derived from marbling adipocytes there is functional enrichment for components of the cytoskeletal architecture (*CNN1*, *ACTA2*, *MYH11*, *ACTB*, *ACTRT2*, *SORBS2*, *KRT18*). Other genes of interest include a) *CH25H* which encodes an enzyme that catalyses the production of a particular oxysterol metabolite b) the microRNA *MIR145* and *c) CTPS2* which catalyses the production of CTP, a high energy analog to ATP but whose hydrolysis is coupled to a restricted subset of metabolic reactions including glycerophospholipid synthesis. Similarly, querying the full list for those genes whose expression > 1.68 fold high in IMF than the other fat depots combined and also higher in IMF than LD by any value yields a list of 76 genes (Additional file 1). There is no functional enrichment for ‘muscle systems process.’

Moreover, in another analysis directed at the specific IMF versus SC comparison, we reported those genes > 2 fold higher expression in IMF than SC (without considering expression in the other fat depots), and higher expression in IMF than LD (by any amount). This produced a list of 73 genes (Additional file 1) including those encoding proteins in extracellular matrix organisation (*GFAP*, *COL28A1*, *COL2A1*, *ITGA8*, *TNC*, *SNCA*, *MYH11*, *MKX*, *COL4A6* and *TNFRSF11B*). Manual curation of the gene list highlighted 3 additional functional groups of interest that are relatively upregulated in IMF versus SC: cholesterol metabolism (*PMP2*, *CH25H*, *CYP4B1*), retinoic acid metabolism (*STRA6*, *MEST*) and insulin and carbohydrate metabolism (*GRB14*, *NR4A3* and *MOXD1*). The expression profiles for the genes within these three functional groupings are shown in Table 4.

**Table 4:** Gene expression patterns of those genes identified by multiple criteria (>2 fold higher expression in IMF than SC and also higher expression in IMF than LD) that encode proteins involved in cholesterol metabolism, retinoic acid metabolism and carbohydrate metabolism. *CH25H* not shown here as its expression profile is documented in Table 3 (all values are log2 normalised mean expression). *P* values are reported for both DE and PIF (based on the SC versus IMF comparison)

Gene	Probe	LD	IMF	SC	Inter	Kid	Omen	Function	*P* value (DE / PIF)
*PMP2*	A_73_110378	3.74	5.44	3.90	3.78	3.74	3.72	Alias *FABP8.* Cholesterol binding. Found in cytoplasm, extracellular exosomes and myelin sheath	0.0000138 / 0.0643
*CYP4B1*	A_73_P033761	7.54	8.12	7.05	8.30	7.36	7.89	Member of the cytochrome p450 superfamily that synthesises cholesterol, steroids and other lipids. Found in the endoplasmic reticulum.	0.00238 / 0.0396
*STRA6*	A_73_106763	4.22	5.33	3.99	4.37	4.21	5.27	Membrane protein involved in the metabolism of retinol involved in numerous developmental processes in many tissues.	0.000154 / 0.0969
*MEST*	A_73_100042	7.27	7.79	6.62	7.25	6.87	7.06	Regulation of lipid storage and response to retinoic acid. Found in the endoplasmic reticulum, extracellular exosome and membrane.	0.00092 / 0.0335
*GRB14*	A_73_114416	5.13	5.73	4.61	5.04	5.33	5.46	Interacts with insulin receptors and insulin-like growth factor receptors, having an inhibitory effect.	0.00149 / 0.1172
*NR4A3*	A_73_P494813	5.57	6.19	5.11	6.04	5.85	6.08	Member of the steroid thyroid hormone retinoid receptor superfamily.	0.00217 / 0.102
*MOXD1*	A_73_104693	4.16	5.09	4.06	4.44	4.47	4.87	Monoxygenase, localised to the endoplasmic reticulum and cell membrane	0.0034 / 0.177

**Fig. 5:**
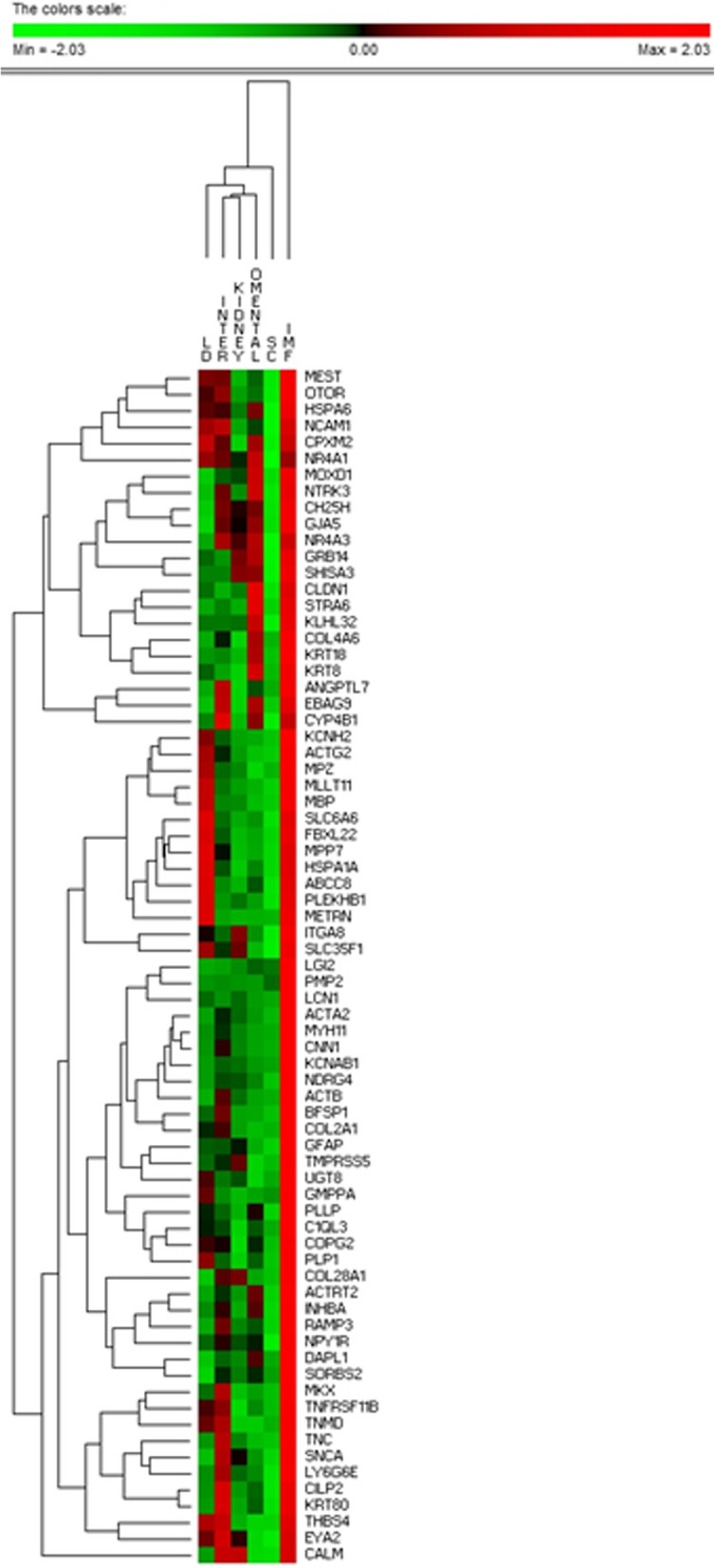
Hierarchical cluster analysis on the 73 genes whose expression suggest particular relevance to the biology of the IMF depot. This list was generated by seeking genes that satisfied two criteria, higher expression in IMF than LD by any amount, and higher expression in IMF than SC by at least 2 fold. The hierarchical clustering on tissues supports the selected gene list as being IMF diagnostic given the first branch in the tree separates the IMF samples from not only the other fat depots but the LD muscle samples too. The clustering on genes identifies positive patterns of co-expression across the treatments

Cluster analysis of the normalised mean expression of the 73 genes across the 5 fat depots and LD muscle indicates the expression of this panel of genes is diagnostic of the IMF depot (Fig. 5). This can be contrasted with the original clustering performed on a randomly selected 10,000 genes whose first branch separates the LD muscle, and not IMF, from all the fat depots. The clustering on rows clusters genes who are co-expressed across tissues. Some of the clusters reflect known functional relatedness, with *KRT8* with *KRT18* reflecting keratin biology and *ACTA2* and *MYH11* reflecting cytoskeletal biology.

Of those 41 genes we identified as more lowly expressed in IMF than SC (by a minimum of 1.32 fold, but unlikely to be due to low expression in the contaminating LD because IMF expression is lower than LD) (Additional file 1) the most extreme 10 are shown in Table 5. There is no functional enrichment for ‘muscle systems process.’

**Table 5:** Top 10 most downregulated genes in IMF versus SC that are probably not attributable to LD contamination (IMF expression lower than LD). All values are log2 normalised mean expression. *P* values are reported for both DE and PIF (based on the SC versus IMF comparison)

Gene	Probe	LD	IMF	SC	Inter	Kid	Omen	*P* value (DE / PIF)
*ANGPTL4*	A_73_101924	14.49	14.41	15.03	14.77	14.67	14.89	0.0168 / 0.00321
*IGFBP5*	A_73_110179	14.65	14.62	15.20	14.79	14.44	14.71	0.0223 / 0.00595
*HOXA10*	A_73_104882	6.99	6.93	8.03	6.28	7.34	5.61	0.000265 / 0.0133
*KLK9*	A_73_117770	8.93	8.44	9.33	8.39	8.15	8.18	0.00197 / 0.0154
*BRE*	A_73_P052356	15.27	15.07	15.56	14.92	15.00	15.02	0.0456 / 0.0216
*PRSS23*	A_73_111432	14.05	14.03	14.53	14.17	13.75	14.18	0.0403 / 0.0166
*C2CD4B*	A_73_P047791	11.58	11.41	12.00	11.43	11.40	12.65	0.0223 / 0.0278
*GPX4*	A_73_106948	14.42	14.29	14.76	14.52	14.62	14.49	0.0485 / 0.0291
*ZCCHC12*	A_73_112381	14.97	14.57	15.02	14.26	14.30	14.43	0.0515 / 0.0294
*FAM71A*	A_73_P048226	4.36	4.18	5.39	4.65	5.12	5.99	0.0000769 / 0.0515

Importantly, none of these refined gene lists generated from our multiple criteria approach yield a significant hypergeometric enrichment for ‘muscle system process.’ This indicates that the multiple criteria thresholding method we have adopted here has successfully eliminated those genes representative of muscle contamination in the IMF sample. The summaries of the number of genes identified by the various single and multiple criteria approaches, and their respective hypergeometric functional enrichments, are found in Tables 6 and 7**,** respectively.

**Table 6:** The numerical and biological outcomes of the single criteria approach we applied to first diagnose the impact of contaminating LD muscle on the IMF samples. The background list was composed of 34,228 genes including duplicates (14,477 unique). A number of these approaches based on high expression in IMF identify the presence of contaminating LD muscle

Single Query	Number of genes satisfying criteria (in brackets duplicates removed)	Functional enrichment for ‘muscle systems process’ (FDR q value)
LD > 4 fold higher than SC	279 (171)	2.04 e-39
IMF > 1.32 fold higher than the average of all fat depots	1833 (1112)	3.05 e-43
IMF > 1.68 fold higher than the average of all fat depots	741 (452)	4.42 e-44
IMF > 2 fold higher than SC	600 (380)	1.26 e-40
IMF > 1.32 fold lower than SC	890 (581)	None identified. Top hit ‘defense response’ 5.09 e-18
IMF > 2 fold higher than LD	329 (219)	None identified. Top hit ‘lipid metabolic process’ 2.98 e-4
IMF > LD by any amount	22,156 (10,679)	None identified. Top hit ‘cell migration’ 1.51 e-11
IMF < LD by any amount	12,072 (6839)	None identified. Top hit ‘translational termination’ 3.02 e-18

**Table 7:** The numerical and biological outcomes of the multiple criteria approach we applied to resolve the impact of contaminating LD muscle on the IMF samples. The background list was composed of 34,228 genes including duplicates (14,477 unique). This approach eliminates the enrichment for ‘muscle systems process’ indicating the impact of the contaminating LD muscle in the IMF samples has been adequately dealt with

Group Query (in brackets number of genes satisfying individual criteria)	Number of genes satisfying criteria (in brackets duplicates removed) / % of total	Deviation from null expectation	Functional enrichment for ‘muscle systems process’ (FDR q value)
IMF > 1.32 fold higher than the average of all fat depots (1833) AND IMF > 2 fold higher than LD (329)	67 (49) / 0.196	3.82 fold enrichment compared to what would be expected if the two criteria were independent	None identified. No other hits at FDR q < 0.01
IMF > 1.68 fold higher than the average of all fat depots (741) AND IMF > LD by any amount (22,156)	105 (76) / 0.307	4.52 fold depletion compared to what would be expected if the two criteria were independent	None identified. No other hits at FDR q < 0.01
IMF > 2 fold higher than SC (600) AND IMF > LD by any amount (22,156)	93 (73) / 0.271	4.87 fold depletion compared to what would be expected if the two criteria were independent	None identified. No other hits at FDR q < 0.01
IMF > 1.32 fold lower than SC (890) AND IMF < LD by any amount (12,072)	49 (41) / 0.143	6.41 depletion compared to what would be expected if the two criteria were independent	None identified. No other hits at FDR q < 0.01

### Hypothesis-driven analysis

#### Lipogenesis in adipocytes

To formally connect these mRNA data to traditional biochemical knowledge, we identified and tabulated the expression profiles of those genes encoding rate-limiting enzymes and other proteins considered influential in the various lipogenic processes (Table 8). This includes the following biochemical processes: precursor transport into the adipocyte cells (glucose and free FA), aspects of intermediate energy metabolism (glycolysis and pyruvate metabolism), de novo FA synthesis, FA elongation, FA desaturation, FA esterification with glycerol and finally the supply of reducing power equivalents.

**Table 8:** Summary of the gene expression patterns of influential enzymes and other proteins in lipogenesis (all data log2 normalised mean expression). *P* values are reported for both DE and PIF (based on the SC versus IMF comparison). Given no additional criteria have been applied here to de-emphasise the effect of LD contamination the *P* values reported in this table do need to be interpreted cautiously. The muscle isoform of PFK (PFKM) is a case in point, as the extreme *P* value awarded is almost certainly a consequence of LD contamination in the IMF samples

Pathway	Pathway flux control enzymes and proteins	Gene	Probe	Cellular localisation	LD	IMF	SC	IMF vs SC FC	IMF minus SC (log2)	*P* value (DE / PIF)
Glucose transport into cell	Facilitated glucose transporter (not an enzyme, stimulated by insulin)	*SLC2A4 (GLUT4)*	A_73_P102656	Membrane	8.08	8.09	7.83	1.19	0.26	0.32 / 0.38
Glycolytic flux	Hexokinase; EC 2.7.1.1	*HK1*	A_73_115982	Mitochondrion	13.31	13.90	14.05	–1.10	–0.14	0.23 / 0.24
		*HK2*	A_73_P082146		10.66	10.38	10.50	–1.08	–0.12	0.26 / 0.29
		*HK3*	A_73_109966		6.63	7.24	7.85	–1.53	–0.61	0.0194 / 0.0883
		*PFKL*	A_73_109643		12.57	13.24	13.51	–1.19	–0.26	0.14 / 0.14
	Phosphofructokinase; EC 2.7.1.11	*PFKM*	A_73_P050301	Cytosol	14.87	14.45	12.38	4.19	2.07	0.0000000049 / 2.68e-10
		*PFKP*	A_73_P118106		9.65	10.45	10.61	–1.12	–0.16	0.22 / 0.26
		*PFKFB1*	A_73_112668		10.77	10.38	9.77	1.53	0.61	0.0689 / 0.10
		*PFKFB2*	A_73_109539		8.03	7.75	7.55	1.15	0.20	0.38 / 0.44
		*PFKFB3*	A_73_P073546		11.70	11.55	11.84	–1.22	–0.29	0.12 / 0.14
		*PFKFB4*	A_73_P500153		15.22	14.81	13.63	2.27	1.18	0.000835 / 0.0000537
		*PKM*	A_73_P100636		10.65	10.10	8.12	3.94	1.98	0.000000022 / 0.0000145
	Pyruvate kinase; EC 2.7.1.105	*PKLR*	A_73_P030011	Cytoplasm, cytosol, mitochondrion	6.28	6.38	6.17	1.16	0.21	0.37 / 0.46
Pyruvate metabolism	Pyruvate carboxylase; EC 6.4.1.1	*PC*	A_73_P426106	Mitochondrion	12.09	12.46	13.20	–1.67	–0.74	0.00723 / 0.00546
	Pyruvate dehydrogenase kinase; EC 2.7.11.2	*PDK1*	A_73_117436	Mitochondrion	6.34	6.37	6.60	–1.18	–0.24	0.168 / 0.27
		*PDK2*	A_73_117896	Mitochondrion	14.14	13.87	13.76	1.08	0.11	0.48 / 0.44
		*PDK3*	A_73_114206	Mitochondrion	10.00	10.43	10.11	1.25	0.32	0.26 / 0.28
		*PDK4*	A_73_119171	Mitochondrion	15.02	14.88	14.41	1.38	0.47	0.14 / 0.0728
	Pyruvate dehydrogenase phosphatase; EC 3.1.3.43	*PDP1*	A_73_110779	Mitochondrion	6.56	6.34	5.83	1.43	0.52	0.11 / 0.29
	Pyruvate dehydrogenase; EC 1.2.4.1	*PDHA1*	A_73_111180	Mitochondrion	16.42	16.09	15.89	1.15	0.20	0.38 / 0.28
Citrate transport out of mitochondria and subsequent conversion into Acetyl CoA	ATP citrate lyase; EC 2.3.3.8	*ACLY*	A_73_111688	Cytoplasm, cytosol, mitochondrion	11.32	11.89	12.12	–1.18	–0.24	0.16 / 0.17
FA synthesis *de novo*	Acetyl CoA carboxylase; EC 6.4.1.2	*ACACA*	A_73_P038926	Cytoplasm, cytosol, mitochondrion	6.13	6.90	7.74	–1.79	–0.84	0.0031 / 0.042
	Fatty acid synthase; EC 2.3.1.85	*FASN*	A_73_P174332	Golgi, cytoplasm, cytosol, mitochondrion	16.08	17.23	17.94	–1.63	–0.71	0.00918 / 0.000528
FA elongation to C18:0	Fatty acid elongase 6; EC 2.3.1.199	*ELOVL6*	A_73_P404726	Endoplasmic reticuluum	9.33	10.83	11.51	–1.61	–0.69	0.0116 / 0.0175
Desaturation to C18:1	Stearoyl-CoA desaturase (delta-9-desaturase); EC 1.14.19.1	*SCD*	A_73_P252739	Endoplasmic reticuluum	14.46	15.33	16.47	–2.20	–1.14	0.000153 / 0.00000163
Supply of reducing power	NADP malate dehydrogenase (Malic enzyme); EC 1.1.1.82	*ME1*	A_73_118619	Cytosol, mitochondrion	11.23	11.50	12.02	–1.43	–0.52	0.0355 / 0.0427
	Glucose-6-phosphate dehydrogenase; EC 1.1.1.49	*G6PD*	A_73_P168952	Cytoplasm, cytosol	6.28	7.11	7.52	–1.33	–0.41	0.0688 / 0.16
	Phosphogluconate dehydrogenase; EC 1.1.1.44	*PGD*	A_73_113428	Cytosol	14.29	15.04	15.63	–1.51	–0.59	0.0223 / 0.0743
	NADP Isocitrate dehydrogenase; EC1.1.1.42 ^b^	*IDH1*								
Esterification ^c^	Glycerol-3-phosphate acyltransferase^a^; EC 2.3.1.15	*GPAM*	A_73_P396821	Mitochondrion, endoplasmic reticulum	12.97	13.71	14.09	–1.3	–0.38	0.0811 / 0.0647
	Diacylglycerol O-acyltransferase 2; EC 2.3.1.20	*DGAT2*	A_73_118582	Endoplasmic reticulum, cytoplasm	14.51	15.86	16.72	–1.82	–0.86	0.00238 / 0.000132
Preformed FA from blood	Lipoprotein lipase; EC 3.1.1.34	*LPL*	A_73_108260	Cell surface membrane	15.27	16.06	16.35	–1.22	–0.29	0.128 / 0.0847
Lipolysis (triacylglycerides to diacylglycerides to monoglycerides)	Hormone sensitive lipase; EC 3.1.1.79	*LIPE*	A_73_101039	Cytosol	10.23	11.16	11.57	–1.33	–0.41	0.0688 / 0.0861
	Adipose triglyceride lipase; EC 3.1.1.3	*PNPLA2*	A_73_P052461	Cytosol, endoplamic reticulum, lipid particle	15.61	15.91	16.30	–1.31	–0.39	0.0768 / 0.0391
	Monoglyceride lipase; EC 3.1.1.23	*MGLL*	A_73_104056	Cytosol, endoplasmic reticuluum	15.19	15.30	15.78	–1.39	–0.48	0.0456 / 0.0203
	Perilipin	*PLIN2*	A_73_P044606	Cytosol, endoplasmic reticulum, lipid particle	12.33	12.63	13.31	–1.61	-0.69	0.0116 / 0.00793
Carrier proteins	Fatty acid binding protein 4 (not an enzyme, quantitative impact on process not known?)	*FABP4*	A_73_113342	Cytoplasm, cytosol	10.69	12.67	13.13	–1.38	-0.46	0.0515 / 0.0469

^a^*GPAM (alias GPAT1)* a quantitatively influential enzyme of esterification according to [14]

^b^Cytosolic NADP isocitrate dehydrogenase (*IDH1*) is not registered as expressed in our fat depot mRNA data

^c^ While not considered rate-limiting for esterification (and glycerolipid synthesis) we wish to draw attention to the expression profiles for a number of additional genes in these two pathways as some have expression profiles indicating higher activity in the IMF than the SC (that cannot be explained by LD muscle contamination) (Table 9)

We can see that some of these canonical lipogenic pathways show clear, consistent patterns of gene expression based on the key enzymes. For example, de novo FA synthesis (*FASN* and *ACACA* 1.63–1.79 fold), FA elongation (*ELOVL6* 1.61 fold), desaturation (*SCD* 2.2 fold), supply of reducing power equivalents (*G6PD*, *ME1* and *PGD* 1.33, 1.43 and 1.51 fold), esterification (*GPAM* and *DGAT2* 1.3 to 1.82 fold) and lipolysis (*PNPLA2*, *LIPE*, *MGLL* and *PLIN2* 1.31, 1.33, 1.39 to 1.61 fold) are rather consistently downregulated in IMF versus SC. On the other hand, there are patterns of both up and downregulation within other pathways. Glycolytic flux and pyruvate metabolism are two such pathways, comprising key genes exhibiting both higher and lower expressed in IMF than SC. The ~ 4 fold upregulation of *PFKM* and *PKM* (i.e. muscle specific isoforms of the enzymes) in IMF versus SC glycolytic flux can most likely be attributed to LD contamination.

We examined a subset of core lipogenic processes in more detail at the whole pathway level. The normalised mean expression data for the FA biosynthesis and FA elongation pathways are shown in Additional file 3. We next manually explored the gene lists, detecting a number of particular isoforms as being upregulated in IMF for esterification and glycerolipid synthesis. These have been tabulated (Table 9) and are contrary to the pathway output (based on *GPAM*, *DGAT*) outlined in Table 8. The expression of *AGPAT9* (esterification) and *MBOAT2* (glycerolipid synthesis) are potentially noteworthy, with a DE in excess of 1.4 fold higher in IMF compared to SC that is not attributable to LD contamination.

**Table 9:** Expression profiles of particular isoforms in esterification and glycerolipid synthesis where IMF shows higher expression than SC that is not attributable to LD contamination. Normalised mean expression values (log2) for LD muscle, dissected IMF and SC. *P* values are reported for both DE and PIF (based on the SC versus IMF comparison). These non significant *P* values need to be interpreted cautiously as these genes do satisfy the independent requirement of higher expression in IMF than LD which we argue makes them potentially noteworthy

Pathway	Enzyme	Gene	Probe	Cellular localisation	LD	IMF	SC	IMF vs SC FC	IMF minus SC (log2)	*P* value (DE / PIF)
Esterification	Glycerol kinase 2; EC 2.7.1.30	*GK2*	A_73_113526	Cytoplasm, mitochondrion	5.83	6.20	5.90	1.23	0.30	0.28 / 0.41
Esterification	Glycerol kinase 5; EC 2.7.1.30	*GK5*	A_73_113437	As above	5.32	5.81	5.47	1.27	0.35	0.24 / 0.39
Esterification	1-acylglycerol-3-phosphate O-acyltransferase 1; EC 2.3.1.51	*AGPAT1*	A_73_110232	Endoplasmic reticuluum	9.67	10.44	10.03	1.33	0.42	0.18 / 0.20
Esterification	1-acylglycerol-3-phosphate O-acyltransferase 4; EC 2.3.1.51	*AGPAT4*	A_73_115012	Endoplasmic reticuluum, membrane	8.07	8.70	8.47	1.17	0.23	0.35 / 0.39
Esterification	1-acylglycerol-3-phosphate O-acyltransferase 9; EC 2.3.1.51	*AGPAT9*	A_73_P272036	Endoplasmic reticuluum, membrane	11.55	12.14	11.63	1.43	0.51	0.11 / 0.10
Glycerolipid synthesis	membrane bound O-acyltransferase domain containing 1; EC 2.3.1.n6	*MBOAT1*	A_73_108662	Endoplasmic reticuluum, membrane	8.57	9.56	9.20	1.28	0.36	0.22 / 0.27
Glycerolipid synthesis	membrane bound O-acyltransferase domain containing 2 EC 2.3.1.n7, 2.3.1.51	*MBOAT2*	A_73_118887	Endoplasmic reticuluum, membrane	5.23	5.68	5.14	1.46	0.55	0.10 / 0.30
Glycerolipid synthesis	Acylglycerol kinase; EC 2.7.1.107, 2.7.1.94	*AGK*	A_73_102007	Mitochondrion	4.28	4.58	4.26	1.25	0.32	0.26 / 0.45

### qRT-PCR

**Fig. 6:**
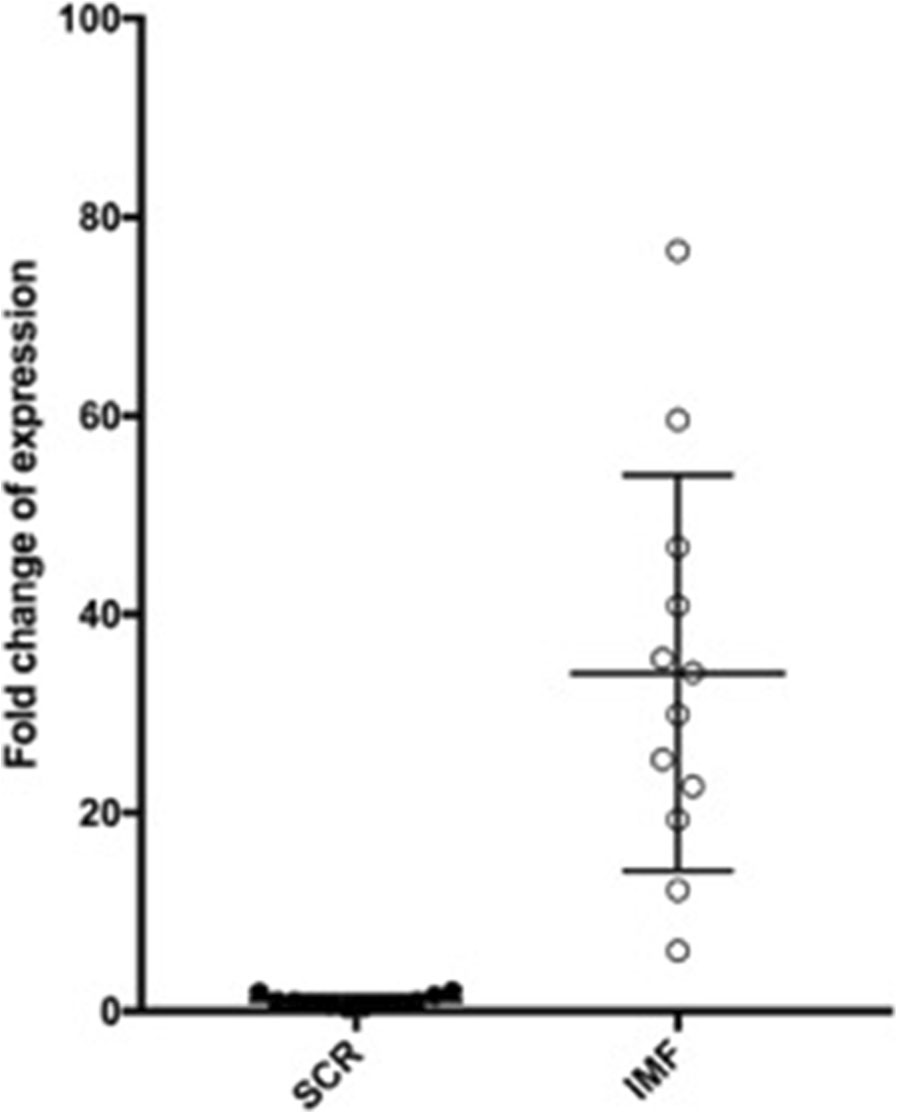
qRT-PCR estimation of *CH25H* mRNA indicates a ~ 36 fold elevation in IMF versus SC fat depots when normalised using RPL as the housekeeping gene

*CH25H* was found to be significantly (*P* < 0.0001; 2 Tailed Mann Whitney U Test) more highly expressed in dissected IMF than SC by ~ 34-fold using the first primer pair (Fig. 6) and 38-fold using the second primer pair, yielding a 36 fold average. The direction of change is the same as for the microarray probe but the absolute difference is ~ 10 fold higher.

### Analysis of LD muscle in Wagyu x Hereford crosses versus Piedmontese x Hereford crosses

**Fig. 7:**
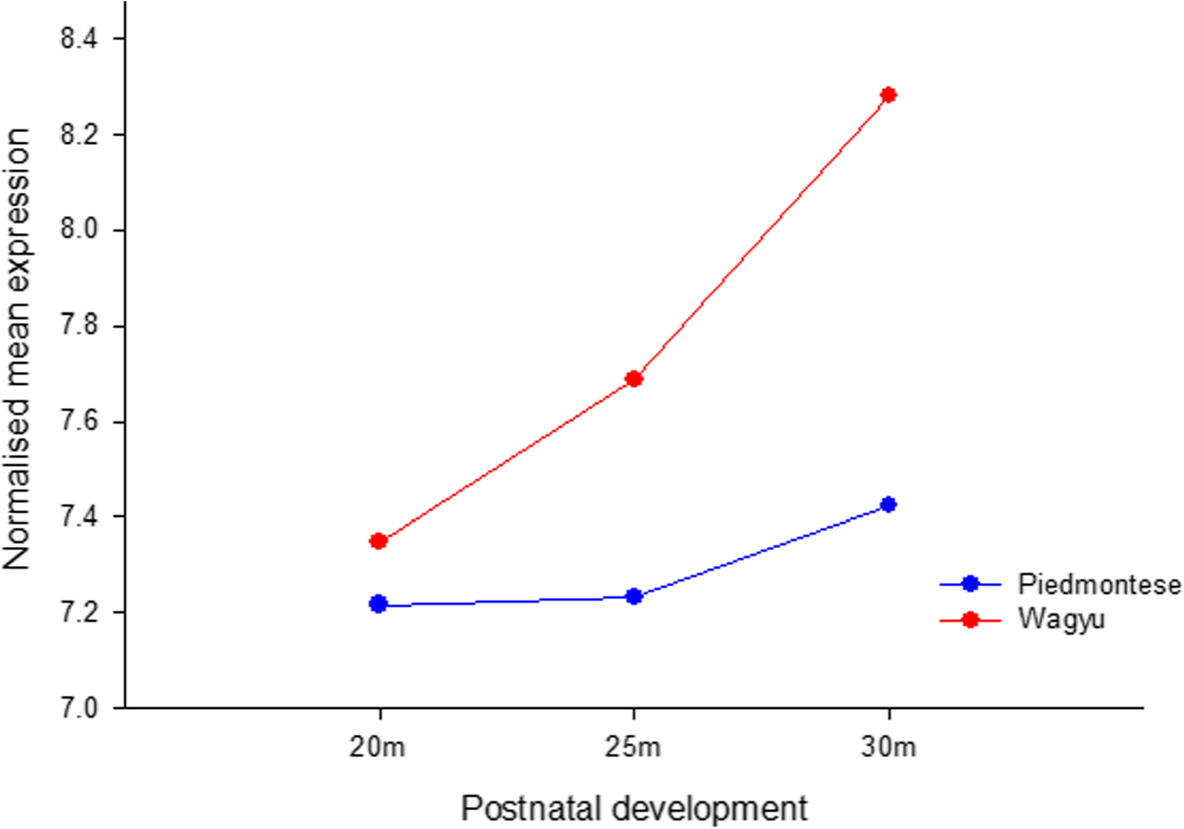
Microarray expression of *CH25H* in postnatal LD muscle increases more rapidly in Wagyu x Hereford animals than Piedmontese x Hereford animals and culminates in a ~ 2 fold difference at 30 months. This ~ 2 fold difference mirrors the observed ~ 2 fold difference in final IMF observed at the anatomical level

The microarray based expression profile for *CH25H* in intact mature postnatal LD muscle is higher in Wagyu x Hereford crosses than Piedmontese x Hereford crosses by 20 months of age, the difference in expression increases with increasing developmental time and by 30 months of age is 2 fold higher (Fig. 7). This 2 fold difference very closely approximates the close to 2 fold difference in carcass IMF previously reported (8.8% IMF in Wagyu x Hereford and 5.1% IMF in Piedmontese x Hereford animals). The increasing significance of the observed differences at 20 m, 25 m and 30 m are reflected by *P* values of 0.478, 0.158 and 0.003 respectively.

### Oxysterol metabolite quantitation

**Fig. 8:**
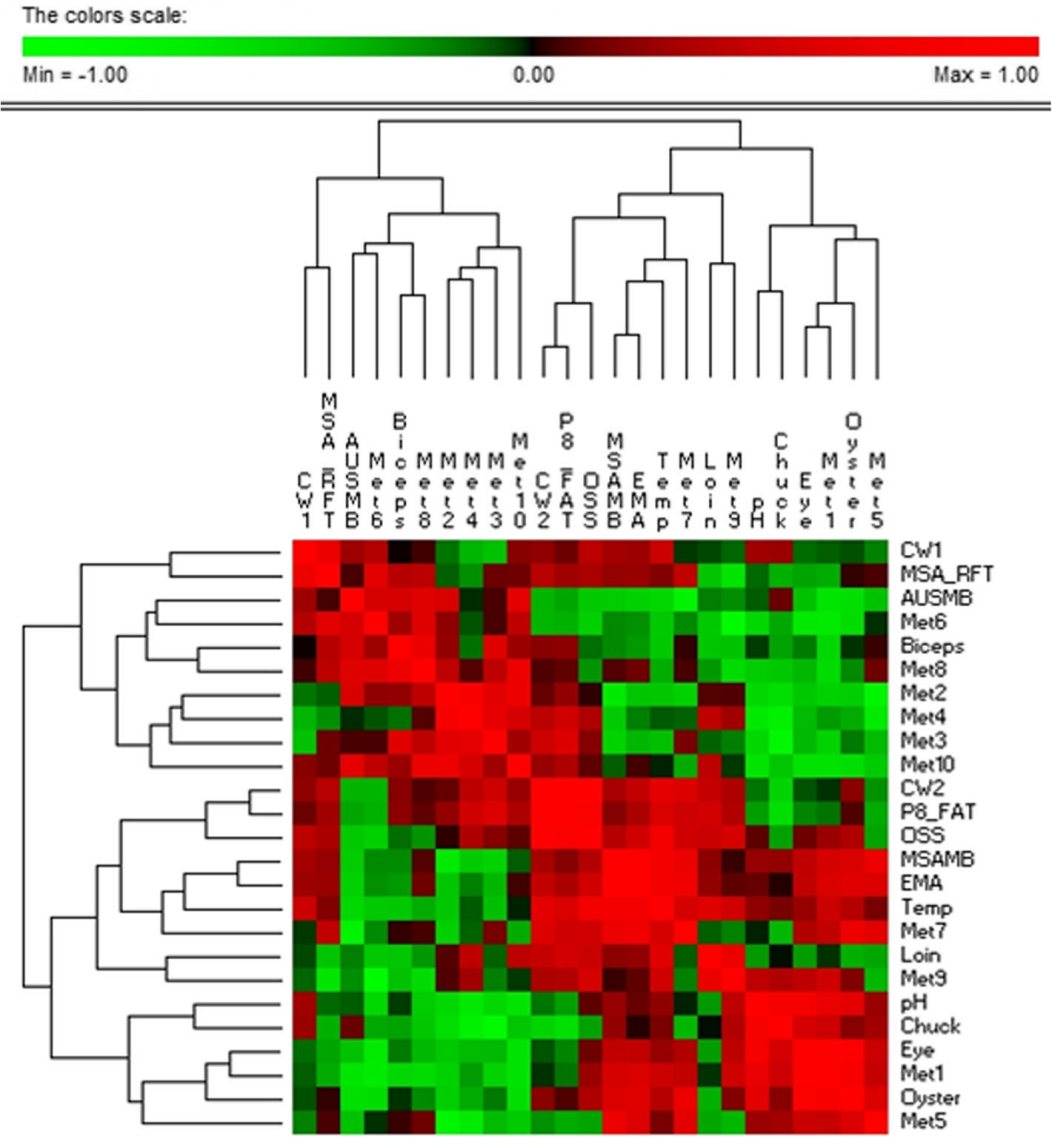
Hierarchical clustering of phenotypes and oxysterol metabolites. This approach clusters (correlations of correlations) on positive relationships only. The metabolite with the tightest positive cluster to a phenotype is metabolite 1 (24S-HC) and eye round IMF (0.91; *P* < 0.001). While 25-HC is not significantly associated with any phenotype, 25-HC does cluster with the metabolites 26-HC and 7-OC which are in turn both correlated with chuck IMF. The key connecting metabolite number to metabolite name is documented in Table 10

The relationships of the oxysterols to the IMF phenotypes are documented below in Table 10 and Fig. 8. The Full SAS output to all 15 phenotypes can be found in Additional file 4.

**Table 10:** Significant correlations at the *P* < 0.05 threshold. Metabolite 7 is also significantly correlated with eye muscle area (0.72, 0.04), the only non-marbling fat association we could detect in comparing all oxysterol metabolites to all carcass phenotypes

ID	Oxysterol systematic name (abbreviation, systematic name)	Biceps IMF	Chuck IMF	Eye Round IMF	Loin IMF	Oyster IMF	MSA MB	AUS MB
Metabolite 1	24S-Hydroxycholesterol (24S-HC, Cholest-5-ene-3β,24S-diol)			0.91, 0.001		0.84, 0.008		
Metabolite 2	25-Hydroxycholesterol (25-HC, Cholest-5-ene-3β,25-diol)							
Metabolite 3	(25R)26-Hydroxycholesterol (26-HC, Cholest-5-en-3β,(25R)26-diol)		−0.83, 0.010					
Metabolite 4	7-Oxocholesterol (7-OC, 3β-Hydroxycholest-5-en-7-one)		−0.73, 0.039					
Metabolite 5	7α-Hydroxycholest-4-en-3-one (7α-HCO)						0.71, 0.049	
Metabolite 6	7α-Hydroxycholesterol (7α-HC, Cholest-5-en-3β,7α-diol)							
Metabolite 7	7α,25-Dihydroxycholest-4-en-3-one (7α,25-diHCO)							−0.74, 0.032
Metabolite 8	7α,25-Dihydroxycholesterol (7α,25-diHC, Cholest-5-ene-3β,7α,25-triol)	0.71, 0.049						
Metabolite 9	7α,26-Dihydroxycholest-4-en-3-one (7α,26-diHCO)							
Metabolite 10	7α,26-Dihydroxycholesterol (7α,26-diHC, Cholest-5-ene-3β,7α,26-triol)

Despite most of the phenotypes provided (8/15) being non-marbling related, at the *P* < 0.05 threshold, the only phenotypes significantly associated with the various oxysterols are marbling related phenotypes (the one exception being 7α,25-dihydroxycholesterol and Eye Muscle Area with r = 0.72, *P* = 0.04)**.** This means the non-IMF fat depot phenotypes did not reach significance with any of the quantitated oxysterols.

We have detected 4 positive correlations and 3 negative correlations. In terms of absolute correlations to IMF phenotypes, 24S-hydroxycholesterol’s relationship to Eye Round IMF is the top performer (r = 0.91; *P* < 0.001) (Additional file 4). No significant relationship to loin IMF was detected for any of the oxysterols although 7α,26-diHCO approached significance (r = 0.67; *P* = 0.0646). Finally, 25-hydroxycholesterol is detected at much higher levels in cattle plasma than in human plasma, consistent with our prediction that the metabolite is largely derived from IMF and humans are essentially a zero IMF species.

## Discussion

### Ruminant fat metabolism

In ruminants the adipocytes are the primary lipogenic site. Consequently, we have focussed our study on the metabolic properties of the various fat depots. Within a ruminant adipocyte, a number of biochemical processes play a role in taking the basic metabolic building blocks (namely pre-formed FA, acetate, D-3 hydroxybutyrate and glucose) from the circulation and converting them into mature TAG. Using a genome-wide transcriptome approach we find evidence for coordinate downregulation of lipogenesis in IMF compared to SC in line with expectation. For example, de novo FA synthesis (*FASN* and *ACACA* 1.63–1.79 fold), FA elongation (*ELOVL6* 1.61 fold), desaturation (*SCD* 2.2 fold), supply of reducing power (*G6PD*, *ME1* and *PGD* 1.33, 1.43 and 1.51 fold) and esterification (*GPAM* and *DGAT2* 1.3 to 1.82 fold) are rather consistently downregulated in IMF versus SC. However, elevated expression of *MBOAT2* and *AGPAT9* does complicate the picture for our understanding of esterification and glycerolipid synthesis in IMF.

In a given pathway we find that the rate limiting enzyme is sometimes among the member(s) of the pathway subject to the highest DE (e.g. *FASN* and *ACACA* in FA synthesis and *ELOVL6* in elongation). Intriguingly, a number of metabolic pathways (lipolysis, pyruvate metabolism, pentose phosphate pathway) have complex patterns of up and down regulated genes between IMF and SC which are challenging to interpret. The major intracellular organelles involved in regulating this collection of lipogenic processes include the endoplasmic reticulum, the peroxisome and the mitochondria and we observe patterns of differential expression associated with all 3 organelles. For example, mitochondrial *TDH* is strongly downregulated in IMF versus SC despite most mitochondrial mRNA for which we have data tending to be elevated in the IMF depot. *TDH* encodes the enzyme threonine dehydrogenase which converts the amino acid threonine into pyruvate during catabolism.

### All fat depots gene expression comparison

All 5 fat depots could be clearly discriminated by the genome-wide gene expression patterns, with the fat depot variation substantially greater than any breed variation within a depot. This analysis clearly shows that at the molecular level all 5 *bovine* fat depots are biologically quite distinct from each other. However, it is beyond the scope of this article to discuss all depots in detail. Nevertheless, the clustering output does represent a robust quality checking metric, implying the various tissue samples have been dissected, RNA extracted, microarray hybridised and statistically normalised without any major mix-up or substantive analytic error.

IMF was awarded a separate branch from the other 4 fat depots suggesting it is by some margin the most functionally divergent. However, the presence of a small amount of LD muscle in the IMF sample but not the other fat depots cannot be ruled out as the major cause of this clustering. On this note, the muscle contamination in IMF had to be formally accounted for in subsequent analyses aimed at understanding the particular biology of IMF. Of the other 4 fat depots that we are confident contain no muscle contamination at all, SC appears to be the most functionally divergent. Plotting the MA plots of SC versus all the other depots in turn consistently points to high expression of *HOXA10* and low expression of *DLK1* in SC. Analogous gene expression observations have been made previously in the SC depots of humans and rodents and have been attributed to variable proportions of populations of pre-adipocytes [15, 16].

#### IMF versus SC PART 1Basic structural differences

It has been previously established that marbling adipocytes are substantially smaller than SC adipocytes with peak diameters of 104 ± 2 μm and 141 ± 5 μm respectively [6]. For geometric reasons IMF samples will possess more cell membrane per cytoplasmic volume on a per unit tissue basis. This will be reflected in the total RNA used as input for transcriptomes analyses and may explain the presence of a set of membrane proteins (*MPZ, NDRG4, RAMP3, PLP1, KCNH2*) in the list of relatively upregulated genes in IMF versus SC. Adipose tissue in general is well innervated [17]. The presence of several genes highly expressed in nervous tissue (*MPZ, MBP, METRN, PMP2, GFAP*) as upregulated in IMF is consistent with the notion that the IMF depot has a modified and/or denser innervation than the SC depot. Because these findings have accounted for LD contamination, we suggest the result is unlikely to be attributed to the simple presence of motor nerves.

Furthermore, IMF appears to possess a unique cytoskeletal architecture, with the marbling adipocytes expressing substantially higher levels of a subset of cytoskeletal genes (*CNN1, ACTA2, MYH11, ACTG2, ACTB, ACTRT2, SORBS2*). It has been known for some time that cytoskeletal architecture changes substantially during adipocyte differentiation from a structured to disorganised state [18]. In line with our data, *CNN1* encoding calponin 1 was found to be upregulated in the muscle transcriptomes of high IMF Meishan compared to low IMF Landrace pigs [19], and also to be more than 2 fold upregulated in 20 month Wagyu by Hereford crosses compared to Piedmontese by Hereford crosses (data from [20]). Our analysis also suggests that IMF adipocytes are embedded in an extracellular matrix environment that is either structurally distinct (or overall more pronounced) when compared to SC, given elevated expression of these genes encoding various extracellular matrix (ECM) components (*GFAP*, *COL28A1*, *COL2A1*, *ITGA8*, *TNC*, *SNCA*, *MYH11*, *MKX*, *COL4A6* and *TNFRSF11B*).

An additional functional group that appears diagnostic of the IMF depot includes a subset of solute transporters (*SLC6A6* and *SLC3SF1*). From a biomarker perspective we have also identified several genes elevated in IMF that encode proteins that have been connected to human obesity and potentially could be detectable in the plasma; these include *INHBA* and *ANGPTL7*.

#### IMF versus SC part II metabolic biochemistry

It is believed SC adipocytes contain lower levels of saturated fats, such as stearic acid (C18:0), than do IMF adipocytes [7]. Prior work profiling *bovine* fatty acids by gas chromatography and corresponding calculations of desaturase indices are broadly consistent with IMF expressing 2.4 fold less *SCD* (encoding the Δ9 desaturase enzyme) in the present data. Furthermore, marbling fat has been shown in vitro to have a relative preference for glucose versus acetate carbon for de novo FA synthesis compared to SC as measured by radioisotope incorporation into bovine adipose tissue slices [6]. In that study glucose was found to be the primary precursor in IMF (51–76%), whereas acetate was dominant in SC (67–77%). In our present data we note that *ACSS2* encoding acetyl coenzyme A synthetase was expressed at ~ 2 fold higher levels in SC than IMF consistent with a relative preference for acetate in the SC depot.

Furthermore, we wish to point out that *NR4A3* (alias *NOR1*) encoding a nuclear hormone receptor in the steroid-thyroid hormone retinoid superfamily was upregulated more than 2 fold in IMF compared to SC. This transcription factor has been found to augment insulin’s ability to stimulate glucose transport in rodent 3 T3-L1 adipocytes [21]. In fact, *NR4A3* was one of several genes identified that indicate substantive differences in carbohydrate (*GRB14, NR4A3, MOXD1*), retinoic acid (*STRA6, MEST*) and cholesterol (*PMP2, CH25H, CYP4B1*) metabolism when comparing IMF to SC.

Malic enzyme (*ME1*) and ATP citrate lyase (*ACLY*), both presumed missing or unimportant in ruminant adipose tissue based on enzymatic analyses under standard metabolic conditions [22] are apparently reasonably highly expressed in these samples at the messenger RNA level. Surprisingly, we did not detect cytoplasmic NADP-isocitrate dehydrogenase (IDH1) mRNA in our data, which has previously been shown to be important lipogenic player in both depots, and particularly in SC [6].

#### IMF versus SC PART III CH25H and the 25 hydroxylation of cholesterol

A qRT-PCR assay for *CH25H* yielded a ~ 34-fold higher expression in IMF than SC. *CH25H* encodes the enzyme cholesterol 25 hydroxylase which converts cholesterol into the metabolite 25 hydroxycholesterol (25-HC). This metabolite has never previously been linked to marbling fat specifically but is certainly known as a potent regulator of cholesterol metabolism [23, 24], fibroblast-mediated tissue remodelling [25] and inflammation [26] all of which have been considered relevant to adiposity in various biological circumstances [27–29].

The tissue specific gene expression profile of *CH25H* in humans favours soft tissue (which includes adipose tissue) according to the TiGER gene expression database [30] supporting the possibility that adipose tissue may have a disproportionate influence over the systemic expression of this enzyme and its products. The identification of *CH25H* as an enzyme of interest prompted us to re-examine a published time course experiment where intact bovine LD muscle had been sampled in low marbling Piedmontese x Hereford breed crosses versus high marbling Wagyu x Hereford breed crosses over development [20].

Interestingly, between 20 and 30 months when IMF is accumulating most rapidly the *CH25H* expression profile in the Wagyu sired cattle increases at a faster rate than the Piedmontese sired cattle, and ends up ~ 2 fold higher. This final expression difference almost exactly mirrors the close to 2 fold difference in actual IMF content observed between the breed crosses, 8.8% in Wagyu sired animals versus 5.1% in Piedmontese sired animals [31]. In other mammalian species, oxysterols have been shown to be present at detectable levels in the circulation using mass spectrometry approaches. Consequently we became interested in the possibility that circulating oxysterols such as 25-HC might be a) detectable in cattle plasma and b) reflect carcass-wide marbling.

### LC-MS estimation of oxysterol metabolites

To test our hypothesis regarding a potential link between circulating oxysterols and carcass-wide marbling we identified 8 animals displaying variation in IMF for whom we had fresh frozen plasma samples. LC-MS estimation of the 10 oxysterols using the methods of [32] was correlated to 15 carcass phenotypes (of which 7 were IMF related). Intriguingly, we found significant associations at the *P* < 0.05 significance threshold for 5 oxysterols to 8 carcass phenotypes, and 7 of those 8 phenotypes were IMF related. This implies that, despite the relatively small sample size, the IMF correlations we have detected are unlikely to be false positives. It appears that circulating oxysterols could form the basis of a diagnostic blood test for IMF content. Such a test could in principle help enable a decision on whether or not to invest in expensive feedlotting.

## Conclusions

We have characterised gene expression patterns discriminating *bovine* fat depots with a particular focus on IMF versus SC. We found the expression patterns were suggestive of a number of observations previously made using traditional biochemical techniques, notably a generalised reduction in lipogenic activity and an apparent preference for glucose carbon in the IMF depot. A number of new discoveries were made, including expression changes consistent with coordinate modifications in the manner IMF handles cholesterol, retinoic acid and carbohydrate metabolism compared to the other depots. These gene expression signals would have remained hidden from view if we had not corrected for the presence of a small amount of muscle in the dissected IMF, and this correction was in turn made possible only by collective normalisation of the fat depot data with the LD muscle data. Of particular interest was an indication of very elevated activity of the cholesterol 25 hydroxylase enzyme in the IMF depot. A suite of oxysterol metabolites were quantitated in plasma using mass spectrometry and appear to be promising circulating biomarkers for whole carcass IMF content.

## Methods

### Beef cooperative research Centre III marbling and fat distribution experiment

The basic experimental details relating to this animal trial have been previously reported [12]. In brief we studied 15 individual 259 day grain fed Angus, Hereford and Wagyu x Angus steers (*n* = 5 per breed) slaughtered at ~ 26 months of age as part of the larger experiment detailed by [12]. Management was standardised for all individuals during growth on pasture from weaning through long-feedlotting. For the transcriptome analysis of the fat depots we focussed on animals’ pasture-fed only during the immediate post-weaning period, rather than animals that received supplement during the immediate post-weaning period [12].

A total of 10 sires were represented across the 15 individuals, with 3 sires per genotype and only 1 or 2 individuals per sire. Fat depot samples were dissected from each carcass as soon as possible after slaughter. Dissected IMF was derived from the *M. longissimus dorsi* (IMF), with other fat samples derived from inter-muscular fat (Inter), omental fat (Omen), kidney fat (Kid) and SC (over the rump) depots. The longissimus dorsi muscle with IMF in situ (LD) was also sampled. Further LD samples were also included in our analysis from animals slaughtered at an earlier time point (kill 2) representing both supplement fed and pasture fed animals. For this transcriptome analysis of the fat depots per se we focussed on animals pasture-fed only during the immediate post-weaning period.

### RNA extraction, microarray hybridisation and normalisation

Total RNA was phenol chloroform extracted using Trizol (Invitrogen) following the manufacturer’s instructions. RNA yield and purity were determined using spectrophotometry and RNA integrity was determined by agarose gel electrophoresis. For each breed by depot comparison 4 of the potential 5 RNA samples were prioritised for microarray hybridisation on the basis of RNA integrity and purity. The RNA was submitted to the Ramaciotti Institute (Randwick, NSW, Australia) for hybridisation to the 4x44K one colour Agilent bovine array. Data was normalised using a previously described mixed model approach [33] and all data in all samples expressed as log2 values (Additional file 2). Given the hybridisation to the microarray is performed based on a given total amount of input RNA, the expression measurements reported effectively represent mRNA abundance on a per unit total RNA basis. All the normalised gene expression data is publicly available via accession GSE136981 and allows for the recreation of our chosen plots and analyses.

#### Hypothesis-free screen

##### Data driven hierarchical clustering

As part of a quality checking procedure and to gain a high level view of the across tissue relationships, the expression profiles of 10,000 genes chosen at random were imported into PermutMatrix [34] for across-depot cluster analysis, with each breed by depot group being represented (with 4 individual animals used to produce the mean breed by depot values) (Fig. 1). With a large number of rows performance is very slow. This random 10,000 can be considered representative of the full genome-wide data. Figure labels are built from concatenating breed and tissue. Although we only used pasture fed animals from kill 5 for the fat depots gene expression, the LD muscle samples we included here are derived from a combination of pasture and supplement fed animals and 2 different kills. Given this, we included the diet and kill information in the figure labels for completeness. Global relationships between depots based on the molecular data were then determined using unsupervised hierarchical clustering performed on columns for all tissues. LD muscle was included in this analysis as an outgroup.

##### Differential expression (DE) analysis

**SC versus the other fat depots**

For the next set of analyses to contrast the depots we first combined the (breed) data within each depot. This meant each depot value is the mean average of 12 individual samples (*n* = 4 for each of the 3 breeds). Two statistical strategies were used to identify differential expression between IMF and SC. First, we used a standard t test for the differences in means assuming equal variances **(**Additional file 5**)**. Secondly, we used z scores from a modified DE (PIF) that we processed through an inverse normal distribution to obtain 1 tailed *P* values (Additional file 6). In terms of visualisation of the data, all fat depots were contrasted for differential expression (DE) against SC, expressed in the form of Minus Average (MA) rocket plots (Fig. 2). For the remainder of the analyses we mainly focussed on the specific comparison between SC and IMF.

**IMF versus SC**

We quantitated the cellular basis of an anomaly (the atypical triangular shaped protuberance) in the structure of the IMF versus SC MA plot. This was achieved by querying the data using the “=AND” function in Microsoft Excel. We asked for all genes that satisfy the following criteria to be returned: expression > 4 fold higher in LD muscle than in SC. We then visually highlighted those genes (i.e highly expressed in LD versus SC) on the IMF versus SC plot (Fig. 3a). The SC fat depot can be considered representative of a clean (i.e. muscle free) sample.

In order to perform function enrichment analysis we computed a modified DE (called Phenotypic Impact Factor or PIF) which we have previously found to account for the distribution of DE with different abundances. This approach is analogous to binning the data by abundance and prioritising the extreme DE within each bin. We view this as attractive because ranking on simple DE runs the risk of prioritising those mRNA that are lowly abundant and near the detection limit of the microarray platform, which is likely the noisy part of the data distribution. PIF is calculated by multiplying the DE by the average abundance across the treatment of interest [35]. After ranking on PIF one can identify the extreme 1% (145 / 14,476) or 5% (724 / 14,476) which can then be submitted for hypergeometric functional enrichment.

**Mitoproteome**

Next, we aimed to establish whether there was a major intracellular composition change in terms of adipocyte mitochondrial content. To achieve this we downloaded the human mitochondrial proteome (http://mitominer.mrc-mbu.cam.ac.uk/release-3.1/begin.do) and after filtering for duplicates generated a unique list of 1045 nuclear and mitochondrially-encoded genes which encode all known mitochondrial proteins. The derivation of this list of mRNA encoding mitochondrial proteins and its use in a similar biological context has been previously described [36]. We overlaid these mitoproteome genes onto the IMF versus SC MA plot and also estimated the deviation from null equilibrium (i.e. to what extent is the mass of the mRNA expression data skewed upwards or downwards with respect to 0) as a proxy for overall mitochondrial content / activity using the method of binomial distance.

**Structural differences between marbling adipocytes and the other fat depot adipocytes**

In a further attempt to analyse (and also better visualise) the effect of contaminating LD on the dissected IMF gene expression we overlaid the IMF versus SC MA plot with a continuous colour spectrum reflecting genes that are most likely arising from the IMF adipocytes per se and not the contaminating LD muscle. This colour coding was achieved in an objective numerical manner by mapping a continuous colour spectrum to the DE calculated between the intact LD muscle (representing expression from mainly muscle cells) and the dissected IMF samples (reflecting expression from mainly marbling fat cells). This means that on the Y axis (DE) of the MA plot those values above 0 that are coloured yellow / red (higher expression in IMF than LD, as well as higher expression in IMF than SC) and those values below 0 that are coloured purple can be confidently ascribed to gene expression arising from the IMF adipocytes themselves.

In a related set of quantitative criteria based analyses aimed at highlighting IMF expression derived from marbling adipocytes and not contaminating muscle cells, we queried the full data set for those genes whose expression satisfied at least one of two criteria: higher expression in IMF than LD muscle and higher expression in IMF than the other depots. There is no standardised approach in the literature for handling the contamination problem identified here. The exact thresholds used in our case were developed empirically through an exploratory, iterative process.

We systematically explored a number of thresholds and observed which ones produced short, manageable gene lists functionally in line with existing biological knowledge. This particular process yields thresholds that are not formal but we wish to emphasise that the numerical decisions we settled on are a) made across the board and are thus transparent and fair b) reproducible from the original data c) are entirely pragmatic – they serve our stated purpose of eradicating those genes whose expression in IMF is likely derived from the contaminating muscle signal and d) hypergeometric analysis of the target list can then be calculated with respect to the background (whole genome) gene list using the GOrilla webtool.

The multiple criteria queries were performed in Microsoft Excel using the “=AND” function. The 4 queries we have reported were generated as follows: 1) *identify genes likely expressed from myocytes not adipocytes* (expression in LD muscle > 4 fold higher than SC); 2) *identify genes diagnostic of IMF adipocytes compared to all the other fat depots 1* (expression in IMF > 2 fold higher than LD and expression in IMF > 1.32 fold higher than the average expression in the other 4 fat depots); 3) *identify genes diagnostic of IMF adipocytes compared to all the other fat depots 2* (expression in IMF > LD by any amount and expression in IMF > 1.68 fold higher than the average expression in the other 4 fat depots); 4) *identify genes diagnostic of IMF adipocytes compared to SC specifically* (expression in IMF > LD by any amount and expression in IMF is > 2 fold higher than in SC).

The output from the last of these queries (higher expression in IMF than LD, at least 2 fold higher expression in IMF than SC) was used as input for hierarchical cluster analysis in Permut matrix following production of a tab delimited .txt file with as many rows as genes and as many columns as tissues.

Finally, we also identified those genes more lowly expressed in IMF than SC (and probably not derived from the LD contamination) as follows: expression in IMF < LD by any amount and < SC by at least 0.4 units).

#### Hypothesis-driven analysis

##### Lipogenesis in adipocytes

Canonical KEGG pathways (https://www.genome.jp/kegg/pathway.html) were used to define lipogenic genes of interest selecting the *Bos taurus* pathway in all cases. For the purposes of this analysis we developed criteria to retrieve one value per gene as most genes in the microarray platform are represented by more than 1 probe. In many cases the probes show a general agreement in direction and magnitude of change, but this is not always the case. In an attempt to generate a single value per gene we decided to prioritise using the Phenotypic Impact Factor (PIF) metric. PIF is computed by multiplying DE by abundance. It has a number of appealing characteristics, both numerical – such as tracking the distribution of the MA plots and de-emphasising noisy rarely expressed transcripts - and biological, such as clearly identifying gross myofibre composition changes when comparing skeletal muscle samples [35]. This prioritisation produces a file containing unique values for 14,476 genes (Additional file 7). The *Bos taurus* KGML files for “Fatty acid synthesis” (BTA00061), “Fatty acid degradation” (BTA00071), “Adipocyte lipolysis” (BTA04923) “Fatty acid elongation” (BTA00061), “Glycerolipid metabolism” (BTA00561), “Pyruvate metabolism” (BTA00620) and “Pentose phosphate metabolism” (BTA00030) were used as a resource to help us identify the mRNA encoding the relevant proteins in each biochemical pathway.

In an effort to further explore the mRNA associated with canonical pathways relevant to ruminant fat metabolism, a number of lipogenic and lipolytic pathways were identified through literature searches in NCBI Pubmed. The component enzymes and genes of the key pathways were then identified through the KEGG pathway database (http://www.genome.jp/kegg/pathway.html) using *Bos taurus* as the species. Further targeted literature searches were also used to determine which enzymes and other proteins could be considered influential in overall pathway flux, including the rate-limiting enzymes.

#### qRT-PCR

We designed 2 sets of primers for *CH25H* (target gene) and 1 set for *RPLP0* (housekeeper) using Primer 3 and accepting the default settings but forcing the amplicon to be 150–250 bp long. The first primer sequences for *CH25H* were forward ttcagtcgcccttctttcct and reverse gtcatggggaacacgaacac. The second primer sequences for *CH25H* were gcaccatcagaattcgtcccand agccagatgttgacaacgtg. For *RPLP0* the sequences were forward caaccctgaagtgcttgacat and reverse aggcagatggatcagcca. These primer pairs produce amplicons of 208 bp, 153 bp and 227 bp respectively. cDNA from IMF and SC was made from total RNA using Superscript III following the manufacturer’s instructions. Primers were first tested by conventional PCR then estimating product size on a 1.6% agarose gel. qRT-PCR was performed on an Applied Biosystems machine using Sybr Green fluorescence chemistry in 10 ul total reaction volumes. No template controls were included on the reaction plate. 12 individual cDNA samples were quantitated for each of the two tissues of interest i.e. IMF and for SC.

#### Analysis of LD muscle gene expression in Wagyu x Hereford versus Piedmontese x Hereford crosses

We made use of a previously published microarray experiment where LD muscle had been sampled across pre and postnatal development in two breed crosses, one with higher IMF and one with lower IMF and higher muscle mass [20, 35]. The normalised mean expression values for the last 3 postnatal time points (20 months, 25 months and 30 months) were retrieved from the data and the expression profiles for *CH25H* plotted for the breed crosses.

#### Oxysterol metabolite quantitation

Fresh frozen plasma samples from 8 cattle divergent in IMF **(**Additional file 8**)** were subject to LC-MS quantitation of a set of oxysterols as previously described [32]. The following oxysterols were detected and quantitated, 24S-hydroxycholesterol (24S-HC), 25-hydroxycholesterol (25-HC), (25R)26-hydroxycholesterol (26-HC), 7-oxocholesterol (7-OC), 7α-hydroxycholest-4-en-3-one (7α-HCO), 7α-hydroxycholesterol (7α-HC), 7α,25-dihydroxycholest-4-en-3-one (7α,25-diHCO), 7α,25-dihydroxycholesterol (7α,25-diHC), 7α,26-dihydroxycholest-4-en-3-one (7α,26-diHCO), 7α,26-dihydroxycholesterol (7α,26-diHC) (Additional file 9). 25-Hydroxycholesterol is the predicted product of the 25 hydroxylase enzyme encoded by *CH25H*. The oxysterol values were then related to 15 cattle phenotypes for each animal using a correlation analysis performed in SAS.

The 15 phenotypes we assessed can be categorised as follows: 7 are marbling related (Meat Standards Australia Marbling Score = MSA, AUSMARBLE Score = AUS MB, Biceps femoris IMF%, Loin IMF%, Chuck tender IMF%, Eye round IMF%, Oyster blade IMF%) and 8 are non-marbling related. The 8 non marbling related phenotypes can be further broken down as follows: 3 are muscle mass related (LD eye muscle area = EMA, Carcass weight derived from hot sides = W1 and cold sides = CW2), 2 are non-IMF Fat related (Rump SC fat depth = P8, rib fat depth = RFT), 2 are meat quality related (temperature at pH 6 = TEMP, ultimate pH = pH) and 1 reflects physiological maturity through assessment of bone development (ossification score = OSS).

##### Statistical analyses

The microarray data were normalised using a mixed model approach as previously described [33]. We used a number of complementary methods to assess significance of differential expression. Genome-wide patterns of DE between IMF and SC were assigned significance based on two approaches: firstly, DE was assessed using a standard t test assuming equal variances; secondly, a modified DE called PIF (Phenotypic Impact Factor) was z score transformed then those z scores were processed through an inverse normal distribution to obtain 1 tailed *P* values. For the hypothesis-free genome-wide screens we ranked the entire gene lists using criteria of a modified DE (Phenotypic Impact Factor or PIF) which after ranking can then be assessed for functional gene set enrichment using hyper-geometric statistics reported as FDR corrected q values.

We used GOrilla software to perform all the functional enrichments [37, 38]. When using the two list approach in GOrilla, we established a target list using a nominal threshold (extreme 1% or 5%) and used a background list taken from the entire genome-wide data. We also used multiple criteria thresholding to generate target gene lists that satisfied strict requirements such they were unlikely to be derived from muscle contaminating the IMF samples and then independently assessed functional enrichment using GOrilla. The estimate of deviation from equilibrium of the cohort of mRNA encoding the mitoproteome was performed in Excel using the BINOM.DIST function. The oxysterol metabolite quantities were related to 15 cattle phenotypes for each animal using a correlation analysis performed in SAS with a *P* < 0.05 considered statistically significant. The qRT-PCR data was analysed using a 2-tailed Mann Whitney U test.

## Supplementary information

**Additional file 1.** A multitab Excel spreadsheet containing the lists of genes satisfying sets of criteria indicative of their likely cell type (e.g. IMF adipocyte) of origin.

**Additional file 2.** A CSV file containing the log 2 normalised mean expression for all 96 individual tissue samples (3 genotypes, 2 diets for intact LD muscle only, 6 tissues and 4 individual replicates per treatment cell) used in this analysis. (CSV 25668 kb)

**Additional file 3.** A word document containing two supplemental tables of gene expression patterns in two pathways (Fatty acid biosynthesis and Fatty acid elongation) relevant to lipogenesis.

**Additional file 4.** An HTM file containing the SAS correlation output for the metabolites and carcass phenotypes. (HTM 232 kb)

**Additional file 5. **The genome-wide *P* values associated with differential expression between the IMF versus SC depots. Significance was established using t tests assuming equal variance.

**Additional file 6. **The genome-wide *P* values associated with the Phenotypic Impact Factor (PIF) values computed for the IMF versus SC depots. Significance was established from z scores processed through an inverse normal distribution to produce 1 tailed *P* values.

**Additional file 7.** An excel spreadsheet containing the Minus and Average (MA) data to recreate the IMF versus SC MA plot, with values for 14,476 probes (one probe per gene). The extreme 1 and 5% by PIF are also listed here.

**Additional file 8.** The carcass phenotypes for those animals whose plasma was quantitated for oxysterols.

**Additional file 9.** The oxysterol quantitation data for the 8 selected animals.

## Data Availability

The datasets generated and / or analysed during the current study are publicly available in the Gene Expression Omnibus (GEO) (accession number GSE136981). The normalised mean expression data for all samples can be found in Additional file 2 and allow for the recreation of all plots and analyses.

## Abbreviations

AUS MB: AUSMARBLE Score
DE: Differential expression
ECM: Extracellular matrix
IMF: Intramuscular fat
Inter: Intermuscular fat
Kid: Kidney fat
LC-MS: Liquid chromatography-mass spectrometry
LD: Longissimus dorsi
MA: Minus average
MSA MB: Meat Standards Australia marbling score
Omen: Omental fat
PIF: Phenotypic Impact Factor
SC: Subcutaneous fat
