# Supplementary material for: Gene expression identifies metabolic and functional differences between intramuscular and subcutaneous adipocytes in cattle
Source: BMC Genomics. 2020 Jan 28;21:77. doi: 10.1186/s12864-020-6505-4 (PMC6986065; doi:10.1186/s12864-020-6505-4)
Supplement: Supplementary file 3 — Additional file 3. A word document containing two supplemental tables of gene expression patterns in two pathways (Fatty acid biosynthesis and Fatty acid elongation) relevant to lipogenesis. [file 12864_2020_6505_MOESM3_ESM.docx]

**Table S1 Fatty Acid Biosynthesis. Normalised mean expression values (log2) for LD muscle, dissected IMF and SC rump.**

| **Probe** | **Gene** | **Enzyme** | **Cellular localisation** | **LD** | **IMF** | **SC** | **IMF vs SC FC** | **IMF minus SC (log2)** | **Pathway flux control** |
| --- | --- | --- | --- | --- | --- | --- | --- | --- | --- |
| A_73_P038926 | *ACACA* | Acetyl CoA carboxylase; EC 6.4.1.2 | Cytoplasm, mitochondrion | 6.13 | 6.90 | 7.74 | -1.79 | -0.84 | Shared |
| A_73_P454566 | *MCAT* | Malonyl CoA:ACP acyltransferase; EC 2.3.1.39 | Mitochondrion | 6.04 | 5.80 | 5.30 | 1.42 | 0.51 | No |
| A_73_P174332 | *FASN* | Fatty acid synthase; EC 2.3.1.85 | Golgi, cytoplasm, mitochondrion | 16.08 | 17.23 | 17.94 | -1.63 | -0.71 | Shared* |
| A_73_117900 | *ACSL3* | Acyl CoA synthetase long chain family member 3; EC 6.2.1.3 | Golgi, endoplasmic reticuluum, mitochondrion, peroxisome | 6.66 | 6.82 | 6.52 | 1.24 | 0.31 | No |

*In bovine mammary tissue *ACACA* was found not to be rate-limiting because the flux control coefficient was less than unity. 63% of flux control was attributed to *ACACA* and the remaining 37% to *FASN* [46]. The gene expression values for *MCAT* and *ACSL3* are not congruent with the reduced expression observed for *ACACA* and *FASN*.

**Table S2 Fatty acid elongation. Normalised mean expression values (log2) for LD muscle, dissected IMF and SC rump.**

| **Probe** | **Gene** | **Enzyme** | **Cellular localisation** | **LD** | **IMF** | **SC** | **IMF vs SC FC** | **IMF minus SC (log2)** | **Pathway flux control** |
| --- | --- | --- | --- | --- | --- | --- | --- | --- | --- |
| A_73_P458692 | *ACAA2* | Acetyl CoA acyltransferase 2; EC 2.3.1.16 | Mitochondrion | 15.12 | 15.28 | 15.70 | -1.34 | -0.43 | No |
| A_73_P049166 | *HADH* | Hydroxyacyl-CoA dehydrogenase; EC 1.1.1.35 | Cytoplasm, mitochondrion | 14.26 | 14.35 | 14.50 | -1.11 | -0.15 | No |
| A_73_111792 | *HADHA* | Hydroxyacyl-CoA dehydrogenase; EC 1.1.1.211 | Mitochondrion | 14.51 | 14.42 | 14.47 | -1.03 | -0.05 | No |
| A_73_P297046 | *ECHS1* | Enoyl CoA hydratase, short chain, 1; EC 4.2.1.17 | Mitochondrion | 17.13 | 17.19 | 17.60 | -1.34 | -0.42 | No |
| A_73_P040356 | *MECR* | Mitochondrial trans-2-enoyl-CoA reductase; EC 1.3.1.38 | Mitochondrion | 7.91 | 7.79 | 8.08 | -1.22 | -0.29 | No |
| A_73_P118552 | *PPT1* | Palmitoyl-protein thioesterase 1; EC 3.1.2.22 | Golgi, cytoplasm, lysosome | 11.47 | 11.95 | 12.35 | -1.32 | -0.40 | No |
| A_73_P404726 | *ELOVL6* | Fatty acid elongase 6; EC 2.3.1.199 | Endoplasmic reticulum | 9.33 | 10.83 | 11.51 | -1.61 | -0.69 | Rate-limiting* |
| A_73_114330 | *HSD17B12* | Hydroxysteroid (17-beta) dehydrogenase 12; EC 1.1.1.330 | Endoplasmic reticuluum | 10.30 | 10.88 | 11.51 | -1.54 | -0.63 | No |
| A_73_P049066 | *PTPLB* | Protein tyrosine phosphatase-like; EC 4.2.1.134 | Endoplasmic reticuluum | 12.46 | 13.58 | 13.95 | -1.29 | -0.37 | No |
| A_73_P472808 | *TECR* | Trans-2,3-enoyl-CoA reductase; EC 1.3.1.93 | Endoplasmic reticuluum | 11.36 | 11.07 | 10.92 | 1.11 | 0.15 | No |
| A_73_P399211 | *ACOT7* | Acyl-CoA thioesterase 7; EC 3.1.2.2 | Cytoplasm, mitochondrion | 9.56 | 9.47 | 9.22 | 1.19 | 0.25 | No |

**ELOVL6* is considered a rate-limiting enzyme catalysing the elongation of saturated and monounsaturated fatty acids [47].

Here, there is strong congruence in expression profiles, with many of the component enzymes being reduced in IMF in line with the rate limiter *ELOVL6*. Exceptions to this pattern are *TECR* and *ACOT7*.
