# Supplementary material for: Gene expression identifies metabolic and functional differences between intramuscular and subcutaneous adipocytes in cattle
Source: BMC Genomics. 2020 Jan 28;21:77. doi: 10.1186/s12864-020-6505-4 (PMC6986065; doi:10.1186/s12864-020-6505-4)
Supplement: Supplementary file 4 — Additional file 4. An HTM file containing the SAS correlation output for the metabolites and carcass phenotypes. (HTM 232 kb) [file 12864_2020_6505_MOESM4_ESM.htm]

SAS Output


|  |
| --- |
| The SAS System |

  

The CORR Procedure

|  |  |
| --- | --- |
| 25 Variables: | CW1 CW2 P8\_FAT MSA\_RFT AUSMB MSAMB EMA OSS Temp pH Biceps Chuck Eye Loin Oyster Met1 Met2 Met3 Met4 Met5 Met6 Met7 Met8 Met9 Met10 |

  

| Simple Statistics | | | | | | |
| --- | --- | --- | --- | --- | --- | --- |
| Variable | N | Mean | Std Dev | Sum | Minimum | Maximum |
| CW1 | 8 | 226.87500 | 18.26345 | 1815 | 206.00000 | 251.00000 |
| CW2 | 8 | 465.87500 | 17.56976 | 3727 | 437.00000 | 487.00000 |
| P8\_FAT | 8 | 462.87500 | 17.82805 | 3703 | 434.00000 | 487.00000 |
| MSA\_RFT | 8 | 26.12500 | 6.59951 | 209.00000 | 16.00000 | 34.00000 |
| AUSMB | 8 | 15.25000 | 2.65922 | 122.00000 | 12.00000 | 21.00000 |
| MSAMB | 8 | 2.37500 | 0.91613 | 19.00000 | 1.00000 | 4.00000 |
| EMA | 8 | 496.25000 | 107.42938 | 3970 | 360.00000 | 680.00000 |
| OSS | 8 | 87.12500 | 6.24357 | 697.00000 | 79.00000 | 95.00000 |
| Temp | 8 | 2.25000 | 0.46291 | 18.00000 | 2.00000 | 3.00000 |
| pH | 8 | 9.61250 | 1.25861 | 76.90000 | 7.90000 | 11.50000 |
| Biceps | 8 | 5.57375 | 0.04138 | 44.59000 | 5.53000 | 5.64000 |
| Chuck | 8 | 10.98875 | 2.46911 | 87.91000 | 8.11000 | 15.07000 |
| Eye | 8 | 7.44500 | 2.61632 | 59.56000 | 4.31000 | 11.43000 |
| Loin | 8 | 7.71250 | 4.85575 | 61.70000 | 4.80000 | 19.51000 |
| Oyster | 8 | 11.69125 | 4.27507 | 93.53000 | 7.09000 | 19.78000 |
| Met1 | 8 | 16.97000 | 9.17452 | 135.76000 | 8.04000 | 31.69000 |
| Met2 | 8 | 0.80619 | 0.24764 | 6.44951 | 0.41695 | 1.12857 |
| Met3 | 8 | 6.12625 | 1.16516 | 49.01001 | 4.72559 | 8.35597 |
| Met4 | 8 | 10.51853 | 2.82507 | 84.14825 | 6.95203 | 14.33702 |
| Met5 | 8 | 5.22857 | 1.51118 | 41.82855 | 2.69607 | 7.13197 |
| Met6 | 8 | 3.52750 | 1.20762 | 28.21998 | 2.55932 | 5.74528 |
| Met7 | 8 | 1.52966 | 0.58531 | 12.23725 | 0.68239 | 2.47462 |
| Met8 | 8 | 5.60470 | 1.04186 | 44.83757 | 3.90416 | 7.10184 |
| Met9 | 8 | 0.35848 | 0.66678 | 2.86786 | -0.93942 | 1.31341 |
| Met10 | 8 | 6.43366 | 1.50164 | 51.46930 | 3.92424 | 8.04768 |

  

| Pearson Correlation Coefficients, N = 8  Prob > |r| under H0: Rho=0 | | | | | | | | | | | | | | | | | | | | | | | | | |
| --- | --- | --- | --- | --- | --- | --- | --- | --- | --- | --- | --- | --- | --- | --- | --- | --- | --- | --- | --- | --- | --- | --- | --- | --- | --- |
|  | CW1 | CW2 | P8\_FAT | MSA\_RFT | AUSMB | MSAMB | EMA | OSS | Temp | pH | Biceps | Chuck | Eye | Loin | Oyster | Met1 | Met2 | Met3 | Met4 | Met5 | Met6 | Met7 | Met8 | Met9 | Met10 |
| CW1 | |  | | --- | | 1.00000 | |  | | |  | | --- | | 0.21275 | | 0.6130 | | |  | | --- | | 0.11797 | | 0.7809 | | |  | | --- | | 0.71129 | | 0.0479 | | |  | | --- | | 0.25370 | | 0.5443 | | |  | | --- | | 0.27642 | | 0.5075 | | |  | | --- | | 0.24728 | | 0.5549 | | |  | | --- | | 0.37099 | | 0.3656 | | |  | | --- | | 0.44356 | | 0.2710 | | |  | | --- | | 0.26545 | | 0.5252 | | |  | | --- | | 0.00071 | | 0.9987 | | |  | | --- | | 0.24098 | | 0.5653 | | |  | | --- | | -0.11978 | | 0.7775 | | |  | | --- | | -0.05683 | | 0.8937 | | |  | | --- | | -0.06775 | | 0.8734 | | |  | | --- | | -0.09175 | | 0.8289 | | |  | | --- | | -0.14103 | | 0.7390 | | |  | | --- | | -0.39820 | | 0.3285 | | |  | | --- | | -0.33726 | | 0.4140 | | |  | | --- | | -0.16875 | | 0.6896 | | |  | | --- | | 0.33458 | | 0.4179 | | |  | | --- | | -0.03539 | | 0.9337 | | |  | | --- | | 0.04666 | | 0.9126 | | |  | | --- | | -0.10868 | | 0.7978 | | |  | | --- | | 0.19077 | | 0.6509 | |
| CW2 | |  | | --- | | 0.21275 | | 0.6130 | | |  | | --- | | 1.00000 | |  | | |  | | --- | | 0.98551 | | <.0001 | | |  | | --- | | 0.35128 | | 0.3935 | | |  | | --- | | -0.33557 | | 0.4164 | | |  | | --- | | 0.28734 | | 0.4902 | | |  | | --- | | 0.39858 | | 0.3280 | | |  | | --- | | 0.91306 | | 0.0015 | | |  | | --- | | 0.61915 | | 0.1017 | | |  | | --- | | -0.14075 | | 0.7396 | | |  | | --- | | 0.18936 | | 0.6533 | | |  | | --- | | -0.49778 | | 0.2094 | | |  | | --- | | -0.07239 | | 0.8647 | | |  | | --- | | 0.41273 | | 0.3095 | | |  | | --- | | 0.19339 | | 0.6463 | | |  | | --- | | -0.03042 | | 0.9430 | | |  | | --- | | 0.10642 | | 0.8020 | | |  | | --- | | 0.46621 | | 0.2443 | | |  | | --- | | 0.36826 | | 0.3694 | | |  | | --- | | -0.21209 | | 0.6141 | | |  | | --- | | -0.33890 | | 0.4115 | | |  | | --- | | 0.54809 | | 0.1596 | | |  | | --- | | 0.06808 | | 0.8727 | | |  | | --- | | 0.30893 | | 0.4566 | | |  | | --- | | 0.23246 | | 0.5796 | |
| P8\_FAT | |  | | --- | | 0.11797 | | 0.7809 | | |  | | --- | | 0.98551 | | <.0001 | | |  | | --- | | 1.00000 | |  | | |  | | --- | | 0.26606 | | 0.5242 | | |  | | --- | | -0.27949 | | 0.5026 | | |  | | --- | | 0.17821 | | 0.6729 | | |  | | --- | | 0.29882 | | 0.4722 | | |  | | --- | | 0.86646 | | 0.0054 | | |  | | --- | | 0.54094 | | 0.1662 | | |  | | --- | | -0.25458 | | 0.5429 | | |  | | --- | | 0.20017 | | 0.6346 | | |  | | --- | | -0.56657 | | 0.1431 | | |  | | --- | | -0.18004 | | 0.6696 | | |  | | --- | | 0.48454 | | 0.2237 | | |  | | --- | | 0.06832 | | 0.8723 | | |  | | --- | | -0.13435 | | 0.7511 | | |  | | --- | | 0.21606 | | 0.6073 | | |  | | --- | | 0.55124 | | 0.1567 | | |  | | --- | | 0.47925 | | 0.2295 | | |  | | --- | | -0.30040 | | 0.4697 | | |  | | --- | | -0.32429 | | 0.4332 | | |  | | --- | | 0.45914 | | 0.2525 | | |  | | --- | | 0.09462 | | 0.8236 | | |  | | --- | | 0.32890 | | 0.4263 | | |  | | --- | | 0.31427 | | 0.4484 | |
| MSA\_RFT | |  | | --- | | 0.71129 | | 0.0479 | | |  | | --- | | 0.35128 | | 0.3935 | | |  | | --- | | 0.26606 | | 0.5242 | | |  | | --- | | 1.00000 | |  | | |  | | --- | | 0.05495 | | 0.8972 | | |  | | --- | | 0.22742 | | 0.5881 | | |  | | --- | | 0.23651 | | 0.5728 | | |  | | --- | | 0.29426 | | 0.4793 | | |  | | --- | | 0.17536 | | 0.6779 | | |  | | --- | | -0.11545 | | 0.7855 | | |  | | --- | | 0.34328 | | 0.4051 | | |  | | --- | | -0.32104 | | 0.4381 | | |  | | --- | | -0.27713 | | 0.5064 | | |  | | --- | | -0.41036 | | 0.3126 | | |  | | --- | | 0.02759 | | 0.9483 | | |  | | --- | | -0.30177 | | 0.4676 | | |  | | --- | | -0.09632 | | 0.8205 | | |  | | --- | | 0.12105 | | 0.7752 | | |  | | --- | | -0.20630 | | 0.6240 | | |  | | --- | | 0.05935 | | 0.8890 | | |  | | --- | | 0.59295 | | 0.1213 | | |  | | --- | | 0.39486 | | 0.3330 | | |  | | --- | | 0.36236 | | 0.3777 | | |  | | --- | | -0.61806 | | 0.1024 | | |  | | --- | | 0.12248 | | 0.7726 | |
| AUSMB | |  | | --- | | 0.25370 | | 0.5443 | | |  | | --- | | -0.33557 | | 0.4164 | | |  | | --- | | -0.27949 | | 0.5026 | | |  | | --- | | 0.05495 | | 0.8972 | | |  | | --- | | 1.00000 | |  | | |  | | --- | | -0.45446 | | 0.2579 | | |  | | --- | | -0.47631 | | 0.2328 | | |  | | --- | | -0.43237 | | 0.2847 | | |  | | --- | | -0.52223 | | 0.1843 | | |  | | --- | | -0.08643 | | 0.8387 | | |  | | --- | | 0.50953 | | 0.1971 | | |  | | --- | | 0.11733 | | 0.7820 | | |  | | --- | | -0.39711 | | 0.3300 | | |  | | --- | | -0.16147 | | 0.7025 | | |  | | --- | | -0.57016 | | 0.1400 | | |  | | --- | | -0.61899 | | 0.1018 | | |  | | --- | | 0.52595 | | 0.1806 | | |  | | --- | | 0.05649 | | 0.8943 | | |  | | --- | | -0.02157 | | 0.9596 | | |  | | --- | | -0.39662 | | 0.3307 | | |  | | --- | | 0.49908 | | 0.2080 | | |  | | --- | | -0.74863 | | 0.0326 | | |  | | --- | | 0.56854 | | 0.1414 | | |  | | --- | | -0.20411 | | 0.6278 | | |  | | --- | | 0.61081 | | 0.1077 | |
| MSAMB | |  | | --- | | 0.27642 | | 0.5075 | | |  | | --- | | 0.28734 | | 0.4902 | | |  | | --- | | 0.17821 | | 0.6729 | | |  | | --- | | 0.22742 | | 0.5881 | | |  | | --- | | -0.45446 | | 0.2579 | | |  | | --- | | 1.00000 | |  | | |  | | --- | | 0.94530 | | 0.0004 | | |  | | --- | | 0.34029 | | 0.4095 | | |  | | --- | | 0.75794 | | 0.0293 | | |  | | --- | | 0.23075 | | 0.5824 | | |  | | --- | | -0.19312 | | 0.6468 | | |  | | --- | | 0.22065 | | 0.5995 | | |  | | --- | | 0.39069 | | 0.3386 | | |  | | --- | | 0.19758 | | 0.6391 | | |  | | --- | | 0.54408 | | 0.1633 | | |  | | --- | | 0.50378 | | 0.2031 | | |  | | --- | | -0.65957 | | 0.0752 | | |  | | --- | | -0.49013 | | 0.2176 | | |  | | --- | | -0.43629 | | 0.2798 | | |  | | --- | | 0.70877 | | 0.0490 | | |  | | --- | | -0.19411 | | 0.6451 | | |  | | --- | | 0.68580 | | 0.0604 | | |  | | --- | | 0.07080 | | 0.8677 | | |  | | --- | | 0.03889 | | 0.9272 | | |  | | --- | | -0.09292 | | 0.8268 | |
| EMA | |  | | --- | | 0.24728 | | 0.5549 | | |  | | --- | | 0.39858 | | 0.3280 | | |  | | --- | | 0.29882 | | 0.4722 | | |  | | --- | | 0.23651 | | 0.5728 | | |  | | --- | | -0.47631 | | 0.2328 | | |  | | --- | | 0.94530 | | 0.0004 | | |  | | --- | | 1.00000 | |  | | |  | | --- | | 0.47149 | | 0.2382 | | |  | | --- | | 0.76843 | | 0.0259 | | |  | | --- | | 0.10605 | | 0.8026 | | |  | | --- | | -0.24060 | | 0.5660 | | |  | | --- | | 0.01641 | | 0.9692 | | |  | | --- | | 0.39301 | | 0.3355 | | |  | | --- | | 0.21667 | | 0.6063 | | |  | | --- | | 0.58215 | | 0.1300 | | |  | | --- | | 0.53635 | | 0.1706 | | |  | | --- | | -0.42285 | | 0.2966 | | |  | | --- | | -0.35287 | | 0.3912 | | |  | | --- | | -0.16531 | | 0.6956 | | |  | | --- | | 0.55458 | | 0.1537 | | |  | | --- | | -0.20802 | | 0.6211 | | |  | | --- | | 0.72215 | | 0.0431 | | |  | | --- | | 0.12296 | | 0.7718 | | |  | | --- | | 0.09376 | | 0.8252 | | |  | | --- | | 0.04920 | | 0.9079 | |
| OSS | |  | | --- | | 0.37099 | | 0.3656 | | |  | | --- | | 0.91306 | | 0.0015 | | |  | | --- | | 0.86646 | | 0.0054 | | |  | | --- | | 0.29426 | | 0.4793 | | |  | | --- | | -0.43237 | | 0.2847 | | |  | | --- | | 0.34029 | | 0.4095 | | |  | | --- | | 0.47149 | | 0.2382 | | |  | | --- | | 1.00000 | |  | | |  | | --- | | 0.77849 | | 0.0229 | | |  | | --- | | 0.08703 | | 0.8376 | | |  | | --- | | -0.12371 | | 0.7704 | | |  | | --- | | -0.26548 | | 0.5251 | | |  | | --- | | 0.12563 | | 0.7669 | | |  | | --- | | 0.44137 | | 0.2736 | | |  | | --- | | 0.31004 | | 0.4549 | | |  | | --- | | 0.24221 | | 0.5633 | | |  | | --- | | 0.02440 | | 0.9543 | | |  | | --- | | 0.17847 | | 0.6724 | | |  | | --- | | 0.31621 | | 0.4454 | | |  | | --- | | -0.27699 | | 0.5066 | | |  | | --- | | -0.46286 | | 0.2481 | | |  | | --- | | 0.47756 | | 0.2314 | | |  | | --- | | -0.22170 | | 0.5977 | | |  | | --- | | 0.49880 | | 0.2083 | | |  | | --- | | 0.09301 | | 0.8266 | |
| Temp | |  | | --- | | 0.44356 | | 0.2710 | | |  | | --- | | 0.61915 | | 0.1017 | | |  | | --- | | 0.54094 | | 0.1662 | | |  | | --- | | 0.17536 | | 0.6779 | | |  | | --- | | -0.52223 | | 0.1843 | | |  | | --- | | 0.75794 | | 0.0293 | | |  | | --- | | 0.76843 | | 0.0259 | | |  | | --- | | 0.77849 | | 0.0229 | | |  | | --- | | 1.00000 | |  | | |  | | --- | | 0.21455 | | 0.6099 | | |  | | --- | | -0.42880 | | 0.2891 | | |  | | --- | | 0.14655 | | 0.7291 | | |  | | --- | | 0.22765 | | 0.5877 | | |  | | --- | | 0.58502 | | 0.1277 | | |  | | --- | | 0.33621 | | 0.4155 | | |  | | --- | | 0.44435 | | 0.2700 | | |  | | --- | | -0.42675 | | 0.2917 | | |  | | --- | | -0.38395 | | 0.3477 | | |  | | --- | | -0.08528 | | 0.8409 | | |  | | --- | | 0.14665 | | 0.7289 | | |  | | --- | | -0.43369 | | 0.2830 | | |  | | --- | | 0.50226 | | 0.2047 | | |  | | --- | | -0.30341 | | 0.4651 | | |  | | --- | | 0.47409 | | 0.2353 | | |  | | --- | | -0.01235 | | 0.9768 | |
| pH | |  | | --- | | 0.26545 | | 0.5252 | | |  | | --- | | -0.14075 | | 0.7396 | | |  | | --- | | -0.25458 | | 0.5429 | | |  | | --- | | -0.11545 | | 0.7855 | | |  | | --- | | -0.08643 | | 0.8387 | | |  | | --- | | 0.23075 | | 0.5824 | | |  | | --- | | 0.10605 | | 0.8026 | | |  | | --- | | 0.08703 | | 0.8376 | | |  | | --- | | 0.21455 | | 0.6099 | | |  | | --- | | 1.00000 | |  | | |  | | --- | | -0.03943 | | 0.9261 | | |  | | --- | | 0.80323 | | 0.0163 | | |  | | --- | | 0.79731 | | 0.0178 | | |  | | --- | | -0.24040 | | 0.5663 | | |  | | --- | | 0.60887 | | 0.1091 | | |  | | --- | | 0.65582 | | 0.0774 | | |  | | --- | | -0.52249 | | 0.1840 | | |  | | --- | | -0.52536 | | 0.1812 | | |  | | --- | | -0.65308 | | 0.0791 | | |  | | --- | | 0.24058 | | 0.5660 | | |  | | --- | | -0.51392 | | 0.1926 | | |  | | --- | | -0.01483 | | 0.9722 | | |  | | --- | | -0.45267 | | 0.2601 | | |  | | --- | | 0.38190 | | 0.3505 | | |  | | --- | | -0.60448 | | 0.1124 | |
| Biceps | |  | | --- | | 0.00071 | | 0.9987 | | |  | | --- | | 0.18936 | | 0.6533 | | |  | | --- | | 0.20017 | | 0.6346 | | |  | | --- | | 0.34328 | | 0.4051 | | |  | | --- | | 0.50953 | | 0.1971 | | |  | | --- | | -0.19312 | | 0.6468 | | |  | | --- | | -0.24060 | | 0.5660 | | |  | | --- | | -0.12371 | | 0.7704 | | |  | | --- | | -0.42880 | | 0.2891 | | |  | | --- | | -0.03943 | | 0.9261 | | |  | | --- | | 1.00000 | |  | | |  | | --- | | -0.26713 | | 0.5224 | | |  | | --- | | -0.15418 | | 0.7155 | | |  | | --- | | -0.42214 | | 0.2975 | | |  | | --- | | -0.03112 | | 0.9417 | | |  | | --- | | -0.50722 | | 0.1995 | | |  | | --- | | 0.22202 | | 0.5972 | | |  | | --- | | 0.58203 | | 0.1301 | | |  | | --- | | -0.13009 | | 0.7588 | | |  | | --- | | 0.03807 | | 0.9287 | | |  | | --- | | 0.25720 | | 0.5386 | | |  | | --- | | 0.02909 | | 0.9455 | | |  | | --- | | 0.70761 | | 0.0496 | | |  | | --- | | -0.39925 | | 0.3271 | | |  | | --- | | 0.24518 | | 0.5584 | |
| Chuck | |  | | --- | | 0.24098 | | 0.5653 | | |  | | --- | | -0.49778 | | 0.2094 | | |  | | --- | | -0.56657 | | 0.1431 | | |  | | --- | | -0.32104 | | 0.4381 | | |  | | --- | | 0.11733 | | 0.7820 | | |  | | --- | | 0.22065 | | 0.5995 | | |  | | --- | | 0.01641 | | 0.9692 | | |  | | --- | | -0.26548 | | 0.5251 | | |  | | --- | | 0.14655 | | 0.7291 | | |  | | --- | | 0.80323 | | 0.0163 | | |  | | --- | | -0.26713 | | 0.5224 | | |  | | --- | | 1.00000 | |  | | |  | | --- | | 0.50316 | | 0.2037 | | |  | | --- | | -0.00266 | | 0.9950 | | |  | | --- | | 0.19025 | | 0.6518 | | |  | | --- | | 0.45560 | | 0.2566 | | |  | | --- | | -0.56371 | | 0.1456 | | |  | | --- | | -0.83190 | | 0.0104 | | |  | | --- | | -0.73186 | | 0.0390 | | |  | | --- | | 0.26970 | | 0.5183 | | |  | | --- | | -0.28990 | | 0.4861 | | |  | | --- | | -0.34641 | | 0.4006 | | |  | | --- | | -0.42972 | | 0.2880 | | |  | | --- | | 0.32467 | | 0.4327 | | |  | | --- | | -0.42162 | | 0.2981 | |
| Eye | |  | | --- | | -0.11978 | | 0.7775 | | |  | | --- | | -0.07239 | | 0.8647 | | |  | | --- | | -0.18004 | | 0.6696 | | |  | | --- | | -0.27713 | | 0.5064 | | |  | | --- | | -0.39711 | | 0.3300 | | |  | | --- | | 0.39069 | | 0.3386 | | |  | | --- | | 0.39301 | | 0.3355 | | |  | | --- | | 0.12563 | | 0.7669 | | |  | | --- | | 0.22765 | | 0.5877 | | |  | | --- | | 0.79731 | | 0.0178 | | |  | | --- | | -0.15418 | | 0.7155 | | |  | | --- | | 0.50316 | | 0.2037 | | |  | | --- | | 1.00000 | |  | | |  | | --- | | -0.24945 | | 0.5513 | | |  | | --- | | 0.90720 | | 0.0019 | | |  | | --- | | 0.91794 | | 0.0013 | | |  | | --- | | -0.42943 | | 0.2883 | | |  | | --- | | -0.35879 | | 0.3828 | | |  | | --- | | -0.36861 | | 0.3689 | | |  | | --- | | 0.44297 | | 0.2717 | | |  | | --- | | -0.65628 | | 0.0772 | | |  | | --- | | 0.32295 | | 0.4352 | | |  | | --- | | -0.35454 | | 0.3888 | | |  | | --- | | 0.38556 | | 0.3455 | | |  | | --- | | -0.59757 | | 0.1177 | |
| Loin | |  | | --- | | -0.05683 | | 0.8937 | | |  | | --- | | 0.41273 | | 0.3095 | | |  | | --- | | 0.48454 | | 0.2237 | | |  | | --- | | -0.41036 | | 0.3126 | | |  | | --- | | -0.16147 | | 0.7025 | | |  | | --- | | 0.19758 | | 0.6391 | | |  | | --- | | 0.21667 | | 0.6063 | | |  | | --- | | 0.44137 | | 0.2736 | | |  | | --- | | 0.58502 | | 0.1277 | | |  | | --- | | -0.24040 | | 0.5663 | | |  | | --- | | -0.42214 | | 0.2975 | | |  | | --- | | -0.00266 | | 0.9950 | | |  | | --- | | -0.24945 | | 0.5513 | | |  | | --- | | 1.00000 | |  | | |  | | --- | | -0.32046 | | 0.4390 | | |  | | --- | | -0.02888 | | 0.9459 | | |  | | --- | | 0.07580 | | 0.8584 | | |  | | --- | | -0.09845 | | 0.8166 | | |  | | --- | | 0.37877 | | 0.3548 | | |  | | --- | | -0.28358 | | 0.4961 | | |  | | --- | | -0.43241 | | 0.2846 | | |  | | --- | | -0.09134 | | 0.8297 | | |  | | --- | | -0.23361 | | 0.5776 | | |  | | --- | | 0.67809 | | 0.0646 | | |  | | --- | | 0.37137 | | 0.3651 | |
| Oyster | |  | | --- | | -0.06775 | | 0.8734 | | |  | | --- | | 0.19339 | | 0.6463 | | |  | | --- | | 0.06832 | | 0.8723 | | |  | | --- | | 0.02759 | | 0.9483 | | |  | | --- | | -0.57016 | | 0.1400 | | |  | | --- | | 0.54408 | | 0.1633 | | |  | | --- | | 0.58215 | | 0.1300 | | |  | | --- | | 0.31004 | | 0.4549 | | |  | | --- | | 0.33621 | | 0.4155 | | |  | | --- | | 0.60887 | | 0.1091 | | |  | | --- | | -0.03112 | | 0.9417 | | |  | | --- | | 0.19025 | | 0.6518 | | |  | | --- | | 0.90720 | | 0.0019 | | |  | | --- | | -0.32046 | | 0.4390 | | |  | | --- | | 1.00000 | |  | | |  | | --- | | 0.84581 | | 0.0081 | | |  | | --- | | -0.45948 | | 0.2520 | | |  | | --- | | -0.15133 | | 0.7206 | | |  | | --- | | -0.30284 | | 0.4659 | | |  | | --- | | 0.55173 | | 0.1563 | | |  | | --- | | -0.53812 | | 0.1689 | | |  | | --- | | 0.67060 | | 0.0687 | | |  | | --- | | -0.19222 | | 0.6484 | | |  | | --- | | 0.17384 | | 0.6806 | | |  | | --- | | -0.56075 | | 0.1482 | |
| Met1 | |  | | --- | | -0.09175 | | 0.8289 | | |  | | --- | | -0.03042 | | 0.9430 | | |  | | --- | | -0.13435 | | 0.7511 | | |  | | --- | | -0.30177 | | 0.4676 | | |  | | --- | | -0.61899 | | 0.1018 | | |  | | --- | | 0.50378 | | 0.2031 | | |  | | --- | | 0.53635 | | 0.1706 | | |  | | --- | | 0.24221 | | 0.5633 | | |  | | --- | | 0.44435 | | 0.2700 | | |  | | --- | | 0.65582 | | 0.0774 | | |  | | --- | | -0.50722 | | 0.1995 | | |  | | --- | | 0.45560 | | 0.2566 | | |  | | --- | | 0.91794 | | 0.0013 | | |  | | --- | | -0.02888 | | 0.9459 | | |  | | --- | | 0.84581 | | 0.0081 | | |  | | --- | | 1.00000 | |  | | |  | | --- | | -0.48453 | | 0.2237 | | |  | | --- | | -0.49366 | | 0.2138 | | |  | | --- | | -0.24620 | | 0.5567 | | |  | | --- | | 0.41855 | | 0.3021 | | |  | | --- | | -0.67021 | | 0.0690 | | |  | | --- | | 0.39584 | | 0.3317 | | |  | | --- | | -0.54020 | | 0.1669 | | |  | | --- | | 0.46312 | | 0.2478 | | |  | | --- | | -0.59353 | | 0.1209 | |
| Met2 | |  | | --- | | -0.14103 | | 0.7390 | | |  | | --- | | 0.10642 | | 0.8020 | | |  | | --- | | 0.21606 | | 0.6073 | | |  | | --- | | -0.09632 | | 0.8205 | | |  | | --- | | 0.52595 | | 0.1806 | | |  | | --- | | -0.65957 | | 0.0752 | | |  | | --- | | -0.42285 | | 0.2966 | | |  | | --- | | 0.02440 | | 0.9543 | | |  | | --- | | -0.42675 | | 0.2917 | | |  | | --- | | -0.52249 | | 0.1840 | | |  | | --- | | 0.22202 | | 0.5972 | | |  | | --- | | -0.56371 | | 0.1456 | | |  | | --- | | -0.42943 | | 0.2883 | | |  | | --- | | 0.07580 | | 0.8584 | | |  | | --- | | -0.45948 | | 0.2520 | | |  | | --- | | -0.48453 | | 0.2237 | | |  | | --- | | 1.00000 | |  | | |  | | --- | | 0.59727 | | 0.1180 | | |  | | --- | | 0.81799 | | 0.0131 | | |  | | --- | | -0.77600 | | 0.0236 | | |  | | --- | | 0.21169 | | 0.6148 | | |  | | --- | | -0.46497 | | 0.2457 | | |  | | --- | | 0.37757 | | 0.3565 | | |  | | --- | | 0.06735 | | 0.8741 | | |  | | --- | | 0.69670 | | 0.0548 | |
| Met3 | |  | | --- | | -0.39820 | | 0.3285 | | |  | | --- | | 0.46621 | | 0.2443 | | |  | | --- | | 0.55124 | | 0.1567 | | |  | | --- | | 0.12105 | | 0.7752 | | |  | | --- | | 0.05649 | | 0.8943 | | |  | | --- | | -0.49013 | | 0.2176 | | |  | | --- | | -0.35287 | | 0.3912 | | |  | | --- | | 0.17847 | | 0.6724 | | |  | | --- | | -0.38395 | | 0.3477 | | |  | | --- | | -0.52536 | | 0.1812 | | |  | | --- | | 0.58203 | | 0.1301 | | |  | | --- | | -0.83190 | | 0.0104 | | |  | | --- | | -0.35879 | | 0.3828 | | |  | | --- | | -0.09845 | | 0.8166 | | |  | | --- | | -0.15133 | | 0.7206 | | |  | | --- | | -0.49366 | | 0.2138 | | |  | | --- | | 0.59727 | | 0.1180 | | |  | | --- | | 1.00000 | |  | | |  | | --- | | 0.62444 | | 0.0979 | | |  | | --- | | -0.36483 | | 0.3742 | | |  | | --- | | 0.05674 | | 0.8938 | | |  | | --- | | 0.14825 | | 0.7261 | | |  | | --- | | 0.37403 | | 0.3614 | | |  | | --- | | -0.16138 | | 0.7026 | | |  | | --- | | 0.26433 | | 0.5270 | |
| Met4 | |  | | --- | | -0.33726 | | 0.4140 | | |  | | --- | | 0.36826 | | 0.3694 | | |  | | --- | | 0.47925 | | 0.2295 | | |  | | --- | | -0.20630 | | 0.6240 | | |  | | --- | | -0.02157 | | 0.9596 | | |  | | --- | | -0.43629 | | 0.2798 | | |  | | --- | | -0.16531 | | 0.6956 | | |  | | --- | | 0.31621 | | 0.4454 | | |  | | --- | | -0.08528 | | 0.8409 | | |  | | --- | | -0.65308 | | 0.0791 | | |  | | --- | | -0.13009 | | 0.7588 | | |  | | --- | | -0.73186 | | 0.0390 | | |  | | --- | | -0.36861 | | 0.3689 | | |  | | --- | | 0.37877 | | 0.3548 | | |  | | --- | | -0.30284 | | 0.4659 | | |  | | --- | | -0.24620 | | 0.5567 | | |  | | --- | | 0.81799 | | 0.0131 | | |  | | --- | | 0.62444 | | 0.0979 | | |  | | --- | | 1.00000 | |  | | |  | | --- | | -0.66656 | | 0.0710 | | |  | | --- | | -0.07382 | | 0.8621 | | |  | | --- | | -0.10013 | | 0.8135 | | |  | | --- | | 0.07764 | | 0.8550 | | |  | | --- | | 0.26217 | | 0.5305 | | |  | | --- | | 0.51535 | | 0.1912 | |
| Met5 | |  | | --- | | -0.16875 | | 0.6896 | | |  | | --- | | -0.21209 | | 0.6141 | | |  | | --- | | -0.30040 | | 0.4697 | | |  | | --- | | 0.05935 | | 0.8890 | | |  | | --- | | -0.39662 | | 0.3307 | | |  | | --- | | 0.70877 | | 0.0490 | | |  | | --- | | 0.55458 | | 0.1537 | | |  | | --- | | -0.27699 | | 0.5066 | | |  | | --- | | 0.14665 | | 0.7289 | | |  | | --- | | 0.24058 | | 0.5660 | | |  | | --- | | 0.03807 | | 0.9287 | | |  | | --- | | 0.26970 | | 0.5183 | | |  | | --- | | 0.44297 | | 0.2717 | | |  | | --- | | -0.28358 | | 0.4961 | | |  | | --- | | 0.55173 | | 0.1563 | | |  | | --- | | 0.41855 | | 0.3021 | | |  | | --- | | -0.77600 | | 0.0236 | | |  | | --- | | -0.36483 | | 0.3742 | | |  | | --- | | -0.66656 | | 0.0710 | | |  | | --- | | 1.00000 | |  | | |  | | --- | | -0.02373 | | 0.9555 | | |  | | --- | | 0.59811 | | 0.1173 | | |  | | --- | | 0.12857 | | 0.7616 | | |  | | --- | | -0.35180 | | 0.3928 | | |  | | --- | | -0.44605 | | 0.2680 | |
| Met6 | |  | | --- | | 0.33458 | | 0.4179 | | |  | | --- | | -0.33890 | | 0.4115 | | |  | | --- | | -0.32429 | | 0.4332 | | |  | | --- | | 0.59295 | | 0.1213 | | |  | | --- | | 0.49908 | | 0.2080 | | |  | | --- | | -0.19411 | | 0.6451 | | |  | | --- | | -0.20802 | | 0.6211 | | |  | | --- | | -0.46286 | | 0.2481 | | |  | | --- | | -0.43369 | | 0.2830 | | |  | | --- | | -0.51392 | | 0.1926 | | |  | | --- | | 0.25720 | | 0.5386 | | |  | | --- | | -0.28990 | | 0.4861 | | |  | | --- | | -0.65628 | | 0.0772 | | |  | | --- | | -0.43241 | | 0.2846 | | |  | | --- | | -0.53812 | | 0.1689 | | |  | | --- | | -0.67021 | | 0.0690 | | |  | | --- | | 0.21169 | | 0.6148 | | |  | | --- | | 0.05674 | | 0.8938 | | |  | | --- | | -0.07382 | | 0.8621 | | |  | | --- | | -0.02373 | | 0.9555 | | |  | | --- | | 1.00000 | |  | | |  | | --- | | -0.19480 | | 0.6439 | | |  | | --- | | 0.56629 | | 0.1434 | | |  | | --- | | -0.86415 | | 0.0056 | | |  | | --- | | 0.38733 | | 0.3431 | |
| Met7 | |  | | --- | | -0.03539 | | 0.9337 | | |  | | --- | | 0.54809 | | 0.1596 | | |  | | --- | | 0.45914 | | 0.2525 | | |  | | --- | | 0.39486 | | 0.3330 | | |  | | --- | | -0.74863 | | 0.0326 | | |  | | --- | | 0.68580 | | 0.0604 | | |  | | --- | | 0.72215 | | 0.0431 | | |  | | --- | | 0.47756 | | 0.2314 | | |  | | --- | | 0.50226 | | 0.2047 | | |  | | --- | | -0.01483 | | 0.9722 | | |  | | --- | | 0.02909 | | 0.9455 | | |  | | --- | | -0.34641 | | 0.4006 | | |  | | --- | | 0.32295 | | 0.4352 | | |  | | --- | | -0.09134 | | 0.8297 | | |  | | --- | | 0.67060 | | 0.0687 | | |  | | --- | | 0.39584 | | 0.3317 | | |  | | --- | | -0.46497 | | 0.2457 | | |  | | --- | | 0.14825 | | 0.7261 | | |  | | --- | | -0.10013 | | 0.8135 | | |  | | --- | | 0.59811 | | 0.1173 | | |  | | --- | | -0.19480 | | 0.6439 | | |  | | --- | | 1.00000 | |  | | |  | | --- | | 0.05279 | | 0.9012 | | |  | | --- | | -0.17094 | | 0.6857 | | |  | | --- | | -0.30123 | | 0.4684 | |
| Met8 | |  | | --- | | 0.04666 | | 0.9126 | | |  | | --- | | 0.06808 | | 0.8727 | | |  | | --- | | 0.09462 | | 0.8236 | | |  | | --- | | 0.36236 | | 0.3777 | | |  | | --- | | 0.56854 | | 0.1414 | | |  | | --- | | 0.07080 | | 0.8677 | | |  | | --- | | 0.12296 | | 0.7718 | | |  | | --- | | -0.22170 | | 0.5977 | | |  | | --- | | -0.30341 | | 0.4651 | | |  | | --- | | -0.45267 | | 0.2601 | | |  | | --- | | 0.70761 | | 0.0496 | | |  | | --- | | -0.42972 | | 0.2880 | | |  | | --- | | -0.35454 | | 0.3888 | | |  | | --- | | -0.23361 | | 0.5776 | | |  | | --- | | -0.19222 | | 0.6484 | | |  | | --- | | -0.54020 | | 0.1669 | | |  | | --- | | 0.37757 | | 0.3565 | | |  | | --- | | 0.37403 | | 0.3614 | | |  | | --- | | 0.07764 | | 0.8550 | | |  | | --- | | 0.12857 | | 0.7616 | | |  | | --- | | 0.56629 | | 0.1434 | | |  | | --- | | 0.05279 | | 0.9012 | | |  | | --- | | 1.00000 | |  | | |  | | --- | | -0.55061 | | 0.1573 | | |  | | --- | | 0.68556 | | 0.0605 | |
| Met9 | |  | | --- | | -0.10868 | | 0.7978 | | |  | | --- | | 0.30893 | | 0.4566 | | |  | | --- | | 0.32890 | | 0.4263 | | |  | | --- | | -0.61806 | | 0.1024 | | |  | | --- | | -0.20411 | | 0.6278 | | |  | | --- | | 0.03889 | | 0.9272 | | |  | | --- | | 0.09376 | | 0.8252 | | |  | | --- | | 0.49880 | | 0.2083 | | |  | | --- | | 0.47409 | | 0.2353 | | |  | | --- | | 0.38190 | | 0.3505 | | |  | | --- | | -0.39925 | | 0.3271 | | |  | | --- | | 0.32467 | | 0.4327 | | |  | | --- | | 0.38556 | | 0.3455 | | |  | | --- | | 0.67809 | | 0.0646 | | |  | | --- | | 0.17384 | | 0.6806 | | |  | | --- | | 0.46312 | | 0.2478 | | |  | | --- | | 0.06735 | | 0.8741 | | |  | | --- | | -0.16138 | | 0.7026 | | |  | | --- | | 0.26217 | | 0.5305 | | |  | | --- | | -0.35180 | | 0.3928 | | |  | | --- | | -0.86415 | | 0.0056 | | |  | | --- | | -0.17094 | | 0.6857 | | |  | | --- | | -0.55061 | | 0.1573 | | |  | | --- | | 1.00000 | |  | | |  | | --- | | -0.03598 | | 0.9326 | |
| Met10 | |  | | --- | | 0.19077 | | 0.6509 | | |  | | --- | | 0.23246 | | 0.5796 | | |  | | --- | | 0.31427 | | 0.4484 | | |  | | --- | | 0.12248 | | 0.7726 | | |  | | --- | | 0.61081 | | 0.1077 | | |  | | --- | | -0.09292 | | 0.8268 | | |  | | --- | | 0.04920 | | 0.9079 | | |  | | --- | | 0.09301 | | 0.8266 | | |  | | --- | | -0.01235 | | 0.9768 | | |  | | --- | | -0.60448 | | 0.1124 | | |  | | --- | | 0.24518 | | 0.5584 | | |  | | --- | | -0.42162 | | 0.2981 | | |  | | --- | | -0.59757 | | 0.1177 | | |  | | --- | | 0.37137 | | 0.3651 | | |  | | --- | | -0.56075 | | 0.1482 | | |  | | --- | | -0.59353 | | 0.1209 | | |  | | --- | | 0.69670 | | 0.0548 | | |  | | --- | | 0.26433 | | 0.5270 | | |  | | --- | | 0.51535 | | 0.1912 | | |  | | --- | | -0.44605 | | 0.2680 | | |  | | --- | | 0.38733 | | 0.3431 | | |  | | --- | | -0.30123 | | 0.4684 | | |  | | --- | | 0.68556 | | 0.0605 | | |  | | --- | | -0.03598 | | 0.9326 | | |  | | --- | | 1.00000 | |  | |
